# Acute nicotine abstinence amplifies subjective withdrawal symptoms and threat-evoked fear and anxiety, but not extended amygdala reactivity

**DOI:** 10.1371/journal.pone.0288544

**Published:** 2023-07-20

**Authors:** Hyung Cho Kim, Claire M. Kaplan, Samiha Islam, Allegra S. Anderson, Megan E. Piper, Daniel E. Bradford, John J. Curtin, Kathryn A. DeYoung, Jason F. Smith, Andrew S. Fox, Alexander J. Shackman

**Affiliations:** 1 Department of Psychology, University of Maryland, College Park, Maryland, United States of America; 2 Neuroscience and Cognitive Science Program, University of Maryland, College Park, Maryland, United States of America; 3 Department of Psychiatry and Behavioral Sciences, School of Medicine, Johns Hopkins University, Baltimore, Maryland, United States of America; 4 Department of Psychology, University of Pennsylvania, Philadelphia, Pennsylvania, United States of America; 5 Department of Psychological Sciences, Vanderbilt University, Nashville, Tennessee, United States of America; 6 Center for Tobacco Research and Intervention and Department of Medicine, School of Medicine and Public Health, University of Wisconsin—Madison, Madison, Wisconsin, United States of America; 7 School of Psychological Sciences, Oregon State University, Corvallis, Oregon, United States of America; 8 Department of Psychology, University of Wisconsin—Madison, Madison, Wisconsin, United States of America; 9 Department of Psychology, University of California, Davis, California, United States of America; 10 California National Primate Research Center, University of California, Davis, California, United States of America; 11 Maryland Neuroimaging Center, University of Maryland, College Park, Maryland, United States of America; University of Pennsylvania Perelman School of Medicine, UNITED STATES

## Abstract

Tobacco smoking imposes a staggering burden on public health, underscoring the urgency of developing a deeper understanding of the processes that maintain addiction. Clinical and experience-sampling data highlight the importance of anxious withdrawal symptoms, but the underlying neurobiology has remained elusive. Mechanistic work in animals implicates the central extended amygdala (EAc)—including the central nucleus of the amygdala and the neighboring bed nucleus of the stria terminalis—but the translational relevance of these discoveries remains unexplored. Here we leveraged a randomized trial design, well-established threat-anticipation paradigm, and multidimensional battery of assessments to understand the consequences of 24-hour nicotine abstinence. The threat-anticipation paradigm had the expected consequences, amplifying subjective distress and arousal, and recruiting the canonical threat-anticipation network. Abstinence increased smoking urges and withdrawal symptoms, and potentiated threat-evoked distress, but had negligible consequences for EAc threat reactivity, raising questions about the translational relevance of prominent animal and human models of addiction. These observations provide a framework for conceptualizing nicotine abstinence and withdrawal, with implications for basic, translational, and clinical science.

## Introduction

Tobacco smoking is a leading preventable cause of disease, disability, and premature death in the U.S. and abroad [1, 2]. More than 1 in 7 U.S. adults (15.2%) regularly smoke tobacco [3]. The annual economic burden of tobacco smoking has been estimated at more than $600 billion in the U.S. alone [4]. Although the dangers of smoking are clear and most smokers (~68%) want to quit, relapse is common and existing cessation aids are far from curative, underscoring the importance of developing a deeper understanding of the mechanisms that maintain nicotine use in humans [2, 5, 6].

The transition from tobacco use to nicotine dependence is undoubtedly complex and dynamic, encompassing alterations in multiple motivational and self-regulatory mechanisms [7–11]. Among them, there is abundant clinical, experience-sampling, and experimental work demonstrating that anxiety and negative reinforcement mechanisms—smoking to alleviate ‘tension’ and emotional distress—play a central role in nicotine dependence and relapse [12–17]. Acute nicotine abstinence is associated with heightened negative emotions—with the strongest meta-analytic effects evident for anxiety (*d* = .63)—and anxiety serves as a diagnostic criterion for nicotine withdrawal in DSM-5 [18–20]. Daily diary and ecological momentary assessment (EMA) studies paint a similar picture, showing that nicotine abstinence leads to pervasive increases in tonic anxiety and amplified reactivity to social conflict and other daily stressors [21, 22]. The accompanying phasic surges in anxiety and other negative emotions (e.g., anger) are, in turn, associated with heightened risk of relapse during cessation attempts [17, 22–27]. Parallel results have been found in laboratory settings, where experimental stressors trigger nicotine craving and promote consumption [28–30]. In sum, acute nicotine abstinence potently increases both tonic (stressor-independent) and reactive (stressor-dependent) anxiety in ways that promote continued nicotine use, with some experimental research suggesting that hyper-reactivity may be particularly evident for temporally uncertain stressors [16, 31, but see 32]. Despite this progress, the neural circuitry underlying withdrawal-related anxiety has remained unclear, impeding the development of more effective or tolerable biological treatments.

Mechanistic studies in rats and mice motivate the popular hypothesis that withdrawal-related anxiety and stress-induced reinstatement of nicotine use reflect functional alterations in the central extended amygdala (EAc) [2, 9, 10], including the central nucleus of the amygdala (Ce) and neighboring bed nucleus of the stria terminalis (BST) [33, 34]. Among nicotine-dependent rodents, acute deprivation is associated with heightened signs of anxiety across a range of threat assays and defensive behaviors (e.g., shock-probe burying) [35]. Focal perturbation studies show that nicotine deprivation-induced increases in anxiety-related behaviors and stress-induced reinstatement of nicotine use critically depend on specific molecular (e.g., corticotrophin-releasing hormone, CRH; norepinephrine) signaling mechanisms in the EAc (Ce/BST), with overlapping effects evident for other addictive substances [13, 35–38]. Yet the relevance of these tantalizing neurobiological discoveries to the complexities of the human brain and human withdrawal remains unclear. Humans and rodents diverged ~80 million years ago, leading to marked behavioral, genetic, and neurobiological differences between the two species [39–41]. Whether the EAc (Ce/BST) circuitry implicated in rodent models of anxious withdrawal is evolutionarily conserved in human tobacco smokers experiencing acute nicotine abstinence remains unexplored and unknown.

**Fig 1:**
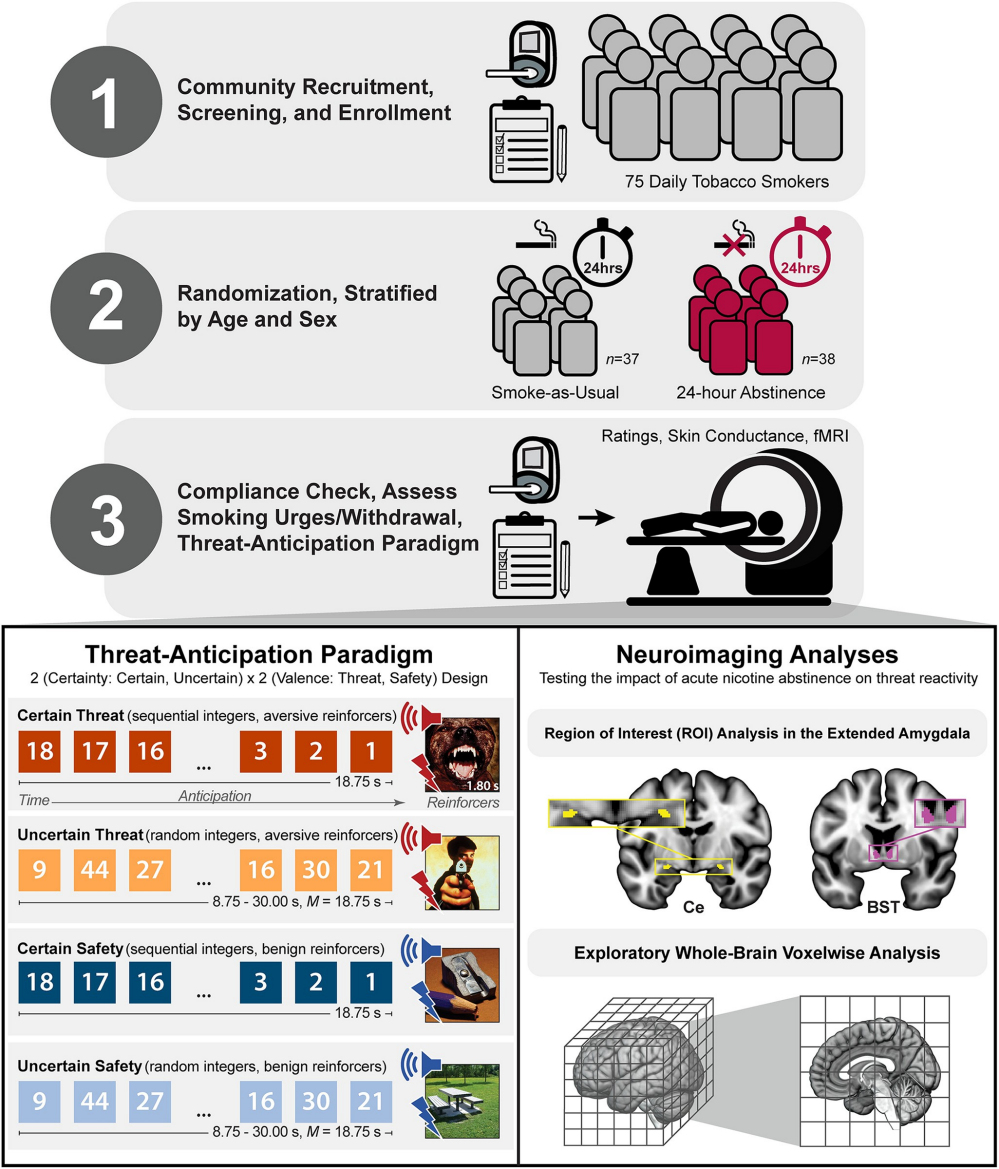
Study overview. ***Top three panels*. (1)** A racially diverse group of 75 daily tobacco smokers was recruited from the community. A multi-stage screening process—including a baseline assessment of breath carbon monoxide (CO) levels—was used to assess eligibility. At the baseline laboratory session, participants completed questionnaire measures of tobacco use and dependence. **(2)** Participants were randomized to either the Smoke-as-Usual or 24-Hour Abstinence groups, stratified by age and sex. **(3)** At the neuroimaging session, protocol compliance was confirmed by breath CO. Prior to scanning, smoking urges and withdrawal symptoms were assessed. During scanning, participants completed a well-established threat-anticipation paradigm that encompassed measures of threat-evoked distress, physiological arousal, and brain function. ***Bottom two panels*. Threat-Anticipation Paradigm**. As shown schematically in the bottom-left panel, the threat-anticipation paradigm took the form of a 2 (*Valence*: Threat/Safety) × 2 (*Temporal Certainty*: Certain/Uncertain) repeated-measures, randomized event-related design. Subjects were completely informed about the task design and contingencies prior to scanning. On Certain Threat trials, subjects saw a descending stream of integers (‘count-down’) for 18.75 s. To ensure robust distress, the anticipation epoch culminated in the delivery of a noxious electric shock, unpleasant photographic image, and thematically related audio clip (e.g., scream). Uncertain Threat trials were similar, but the integer stream was randomized and presented for an uncertain and variable duration (8.75–30.00 s). Participants knew that something aversive was going to occur, but they had no way of knowing precisely *when*. Safety trials were similar, but terminated with the delivery of benign reinforcers (e.g., just-perceptible electrical stimulation). Mean duration of the anticipation epochs was identical across conditions. Subjects were periodically prompted to rate the intensity of fear/anxiety experienced during the anticipation period of the prior trial. Skin conductance was continuously acquired throughout. **Neuroimaging Analyses.** As shown schematically in the bottom-right panel, two approaches were used to test the impact of acute hour nicotine abstinence on neural reactivity to threat. Guided by mechanistic work in animal models, hypothesis testing focused on two well-established, anatomically defined EAc regions-of-interest (ROIs): the Ce (*left*) and BST (*right*). Because the ROIs (i.e., voxel-level measurements) were chosen *a priori*—on the basis of anatomy, rather than suprathreshold threat reactivity—this approach provides unbiased effect size estimates [48]. Standardized regression coefficients were extracted and averaged across voxels for each combination of ROI, threat type (Uncertain/Certain), and participant. Hypothesis testing used a standard mixed-effects general linear model (GLM). For illustrative purposes, 1-mm ROIs are shown. Analyses employed ROIs decimated to the 2-mm resolution of the fMRI data. As shown schematically in the bottom-right panel, exploratory whole-brain voxelwise analyses were also performed. Abbreviations—BST, bed nucleus of the stria terminalis; Ce, central nucleus of the amygdala; fMRI, functional magnetic resonance imaging; hrs., hours; *M*, mean; s, seconds.

Here we used a novel combination of assessments—including questionnaire measures of smoking urges and withdrawal, a well-established certain/uncertain threat-anticipation (‘threat-of-shock’) paradigm, ratings of threat-evoked fear and anxiety, a psychophysiological (skin conductance) measure of threat-evoked arousal, and functional MRI (fMRI)—and a between-subjects randomized trial design to understand the subjective and biological consequences of 24-hour nicotine abstinence in a racially diverse sample of 75 daily tobacco smokers (**Fig 1**). Prior work demonstrates that the threat-anticipation paradigm elicits robust distress and arousal [42, 43]. This stands in stark contrast to popular ‘threat-related’ emotional face fMRI paradigms [44–46], which do not elicit distress in typical adults and are better conceptualized as probes of emotion perception [47]. A best-practices fMRI pipeline enhanced our ability to resolve the Ce and BST, the two major subdivisions of the EAc. Anatomically defined regions-of-interest (ROIs) made it possible to test the central hypothesis that EAc (Ce/BST) threat reactivity would be potentiated in the 24-Hour Abstinence group (vs. Smoke-as-Usual), and to explore the possibility that this anticipated hyper-reactivity would be more evident during the anticipation of temporally Uncertain Threat [31, but see 32]. In contrast to whole-brain voxelwise analyses—which screen thousands of voxels for evidence of statistical significance and yield optimistic effect size estimates in suprathreshold regions—anatomically defined ROIs ‘fix’ the outcomes of interest *a priori*, providing statistically unbiased estimates of brain-phenotype associations [i.e., effect sizes; 48].

Understanding the impact of nicotine abstinence on human EAc reactivity is conceptually and practically important. It affords an opportunity to assess the translational relevance of influential neurobiological models of addiction derived almost exclusively from work in rodents [2]. Successful translation across species would prioritize EAc targets for nicotine-related treatment development and testing [49, 50]. Discrepancies across species would point to the need to refine animal models, human paradigms, or both. Both outcomes would inform the development of bidirectional translational models of nicotine dependence, withdrawal, and relapse. While not a central goal, the present project also promises to shed new light on the neural systems recruited by certain and uncertain threat anticipation, a key question for many fear and anxiety researchers [34, 51–57].

## Method

In this section, we report how we determined our sample size, all data exclusions, all manipulations, and all measures in the study.

### Participants and enrollment criteria

Seventy-eight daily tobacco smokers were enrolled and scanned. Eligibility was determined using a multi-stage procedure that included on-line, telephone, and face-to-face assessments. Eligibility criteria included: 18–40 years old; smoke at least 10 cigarettes/day for at least 6 months; baseline breath carbon monoxide (CO) level of ≥8 parts per million (ppm) at baseline; normal or corrected-to-normal color vision; English proficiency; and self-reported absence of a non-nicotine substance use disorder, lifetime psychotic or bipolar disorder, lifetime neurological or pervasive developmental disorder, very premature birth, current psychiatric treatment, MRI contraindications, or prior experience with aversive electrical stimulation. Similar tobacco-use and carbon-monoxide criteria have been used in prior laboratory studies and clinical trials [e.g. 31, 32, 58, 59]. Ten or more cigarettes per day is considered a ‘moderate’ level of tobacco use [60]. All participants provided informed written consent. Procedures were approved by the University of Maryland, College Park Institutional Review Board (protocol #824438). Data from this project have not been featured in any prior publications.

Three individuals were excluded from all analyses due to study withdrawal (*n* = 1) or inadequate task compliance (*n* = 2; see below). The final sample of 75 participants was racially diverse (*M* = 30.05 years, *SD* = 5.64; 33.3% female; 49.3% African American, 25.3% White Non-Hispanic, 12.0% Asian, 9.4% Multiracial/Other, 4.0% Hispanic or Latino/a), enhancing equity and generalizability [61, 62]. Educational attainment and income data are provided in **S1 and S2 Tables**. As detailed in **Table 1**, the Smoke-as-Usual and 24-Hour Abstinence groups were well matched. The two groups also did not differ in their general engagement with the MTC paradigm, as indexed by the proportion of ratings completed (**Table 1**). Sensitivity analyses performed using Welch’s *t*-test yielded identical conclusions (not reported). All participants showed acceptable levels of head-motion artifact, as detailed below.

**Table 1:** Descriptive statistics for the complete sample, Smoke-as-Usual group, and 24-Hour Abstinence group.

		Complete Sample	Smoke-as-Usual	24-hour Abstinence	Group Differences
**Baseline demographic variables**	***Total N***	75	37	38	N/A
	***Female N***	25	15	10	χ2 = 1.12, *p* = 0.28
	***Age (years)***	30.05 (5.64)	30.11 (5.53)	30 (5.81)	*t*(73) = -0.08, *p* = 0.93
	***Years smoked*, *mean (SD)***	11.89 (6.49)	12.03 (6.86)	11.76 (6.19)	*t*(73) = -0.17, *p* = 0.86
	***Cigarettes/day*, *mean (SD)***	13.47 (4.46)	13.05 (4.58)	13.87 (4.36)	*t*(73) = 0.78, *p* = 0.43
	***FTCD score*, *mean (SD)***	5.71 (1.38)	5.65 (1.44)	5.78 (1.33)	*t*(73) = 0.39, *p* = 0.69
	***WISDM total score*, *mean (SD)***	52.42 (14.56)	50.33 (12.18)	54.46 (16.46)	*t*(73) = 1.23, *p* = 0.22
	***WISDM negative reinforcement subscale score*, *mean (SD)***	4.72 (1.49)	4.67 (1.34)	4.78 (1.64)	*t*(73) = 0.33, *p* = 0.73
	***Baseline CO level (ppm)*, *mean (SD)***	26.4 (14.75)	28.95 (17.63)	23.92 (10.94)	*t*(73) = -1.48, *p* = 0.14
**Neuroimaging session variables**	***WSWS total score*, *mean (SD)***	N/A	36.68 (12.03)	61.97 (18.50)	*t*(73) = 7.00, *p*<0.001
	***WSWS anxiety subscale score*, *mean (SD)***	N/A	5.08 (3.29)	9.29 (3.66)	*t*(73) = 5.23, *p*<0.001
	***BQSU score*, *mean (SD)***	N/A	26.38 (13.03)	54.29 (10.83)	*t*(73) = 10.09, *p*<0.001
	***Experimental CO level (ppm)*, *mean (SD)*** ^***a***^	N/A	35.75 (41.36)	3.35 (5.84)	*t*(73) = -4.78, *p*<0.001
	***Aversive electrical stimulation level (V)*, *mean (SD)***	121.33 (45.14)	121.73 (45.35)	120.95 (45.53)	*t*(73) = -0.07, *p* = 0.94
	***Benign electrical stimulation level (V)*, *mean (SD)***	24.43 (5.48)	24.70 (5.42)	24.16 (5.59)	*t*(73) = -0.42, *p* = 0.66
	***Ratings Completed (%)*, *mean (SD)***	94.95 (9.88)	96.33 (8.52)	93.61 (10.99)	*t*(73) = -1.19, *p* = 0.23

^a^ CO levels at the neuroimaging session were also significantly reduced in the 24-Hour Abstinence group relative to their own baseline levels. *t*(37) = 11.27, *p* < .001. Abbreviations—BQSU, Brief Questionnaire of Smoking Urges; CO, carbon monoxide; FTND, Fagerstrom Test for Cigarette Dependence; ppm, parts per million; *SD*, standard deviation; V, volts; WISDM, Wisconsin Inventory of Smoking Dependence Motives; WSWS: Wisconsin Smoking Withdrawal Scale.

### Power analysis

Sample size was determined *a priori* as part of the application for the grant that supported data collection (R21-DA040717). The target sample size (*n*≈72) was chosen to afford acceptable power and precision given available resources. At the time of study design, Gpower (version 3.1.9.2) indicated 88.3% power to detect a benchmark “large” group mean difference (*d* = .80) with 10% planned attrition (*n* = 32/group; *df* = 63) using α_*two-tailed*_ = .05 [63]. In practice, funds were available to support the enrollment of 78 participants. With the exception of quality assurance checks performed using data from the first few participants, all analyses were performed following the acquisition of the entire dataset. The final sample (n = 75; **Table 1**) was comparable to or larger than many prior studies focused on threat reactivity in acutely abstinent tobacco smokers [31, 32, 64]. A post hoc power analysis indicated that the final sample was powered to detect medium-to-large effects (power >80% for *d*>.66).

### General procedures

#### Recruitment

Daily tobacco smokers from the DC-Baltimore metropolitan region were recruited using a combination of on-line (e.g., posts to social media platforms and groups) and off-line advertisements (e.g., fliers and business cards distributed at high-traffic local restaurants, coffee shops, and libraries). Preliminary eligibility was determined using a multi-stage screening process that included on-line surveys and a telephone screening.

#### Baseline laboratory session

Potentially eligible individuals were invited to a baseline laboratory session. Smoking status was biochemically verified using a Micro+ Smokerlyzer (coVita, Santa Barbara, CA). All participants demonstrated an exhaled CO level of at least 8 ppm, averaged across three serial tests (**Table 1**). Participants also completed a battery of standardized measures of tobacco use and dependence. Participants were then randomly assigned to either the Smoke-as-Usual (SAU) or 24-Hour Abstinence group, stratified by age and sex. The Smoke-as-Usual group was instructed to continue their normal smoking habits prior to their neuroimaging session, whereas the 24-Hour Abstinence group was instructed to refrain from smoking or using any other nicotine products for 24 hours prior to the scheduled neuroimaging session. To encourage protocol compliance, a series of email and text message reminders was sent prior to and during the final 24 hours.

#### Neuroimaging session

Upon arrival at the neuroimaging session, protocol compliance was assessed. For the 24-Hour Abstinence group, participants were allowed to proceed with scanning upon self-reporting nicotine abstinence and receiving a CO reading of <50% of their baseline level. Participants in the Smoke-as-Usual group demonstrated CO levels of at least 8 ppm. By design, measured CO levels were significantly reduced in the 24-Hour Abstinence group (**Table 1**). Prior to scanning, participants were offered a brief break, and those in the Smoke-as-Usual group were encouraged to smoke *ad libitum*. Prior to scanning, participants completed a battery of standardized questionnaires assessing smoking urges and withdrawal symptoms (see below). During scanning, foam inserts were used to immobilize the participant’s head within the head-coil and mitigate potential motion artifact. Participants were continuously monitored using an MRI-compatible eye-tracker (Eyelink 1000; SR Research, Ottawa, Ontario, Canada) and the AFNI real-time motion plugin [65]. Measures of respiration and breathing were continuously acquired during scanning using a respiration belt and photo-plethysmograph affixed to the first digit of the non-dominant hand. Following the last scan, participants were removed from the scanner, debriefed, compensated, and discharged.

### Measures of nicotine use and dependence

#### Fagerstrom Test for Cigarette Dependence (FTCD)

To assess potential group differences in nicotine dependence, participants completed the FTCD (6-items) at the baseline laboratory session [66–68]. Yes-or-no items are rated as 1-or-0. Multiple-choice items are rated from 0 to 3. Higher total (sum) scores indicate greater nicotine dependence.

#### Wisconsin Inventory of Smoking Dependence Motives (WISDM)

To assess potential group differences in general and negative reinforcement-specific tobacco smoking motivation, participants completed the WISDM (68 items) at the baseline laboratory session [69]. Items are rated on a 7-point scale, with 1 indicating ‘not true of me at all’ and 7 indicating ‘extremely true of me’. The total score is the sum of the means across subscales. The total scale (68 items, α = .97) and negative-reinforcement facet scale (6 items, α = .88) were calculated for each participant. Higher scores indicate greater nicotine dependence.

#### Wisconsin Smoking Withdrawal Scale (WSWS)

To assess general and anxiety-specific symptoms of nicotine withdrawal syndrome and to verify protocol compliance, participants completed the WSWS (28 items) at the neuroimaging session [70]. Items are rated on a 5-point scale, with 0 indicating ‘strongly disagree’ and 4 indicating ‘strongly agree’. Items are summed to generate a score for each subscale and a total score. The total scale (28 items, α = .94) and anxiety subscale score (4 items, α = .83) were calculated for each participant. Higher scores indicate greater nicotine withdrawal symptoms.

#### Brief Questionnaire of Smoking Urges (BQSU)

To assess tobacco smoking craving and to verify protocol compliance, participants completed the BQSU (10 items) at the neuroimaging session (West & Ussher, 2009). Items are rated on a 7-point scale, with 1 indicating ‘strongly disagree’ and 7 indicating ‘strongly agree’. Items are summed to generate a score for each subscale and a total score. The total score (10 items, α = .96) was calculated for each participant. Higher scores indicate greater smoking urges/craving.

### Threat-anticipation paradigm

#### Paradigm structure and design considerations

The Maryland Threat Countdown (MTC) is a well-established, fMRI-optimized version of temporally uncertain-threat assays previously validated using fear-potentiated startle and acute anxiolytic administration (e.g., benzodiazepine) in mice [71, 72], rats [73], and humans [74]. The MTC has been successfully used in a number of human fMRI studies [42, 43].

The MTC paradigm (**Fig 1**) takes the form of a 2 (*Valence*: Threat/Safety) × 2 (*Temporal Certainty*: Uncertain/Certain) randomized, event-related, repeated-measures design (3 scans; 6 trials/condition/scan). Subjects were completely informed about the task design and contingencies prior to scanning. Simulations were used to optimize the detection and deconvolution of task-related hemodynamic signals (variance inflation factors <1.54). Stimulus presentation and ratings acquisition were controlled using Presentation software (version 19.0, Neurobehavioral Systems, Berkeley, CA).

Valence was continuously signaled during the anticipation epoch by the background color of the display. Trial certainty was signaled by the nature of the integer stream. On Certain Threat trials, subjects saw a descending stream of integers (‘count-down;’ 30, 29, 28. . .3, 2, 1) for 18.75 s (0.625 s/integer). To ensure robust distress, this anticipation epoch always culminated with the delivery of a noxious electric shock, unpleasant photographic image (e.g., mutilated body), and thematically related audio clip (e.g., scream, gunshot). Uncertain Threat trials were similar, but the integer stream was randomized and presented for an uncertain and variable duration (8.75–30.00 s). Here, participants knew that something aversive was going to occur, but they had no way of knowing precisely *when*. Safety trials were similar, but terminated with the delivery of benign reinforcers (i.e., just-perceptible electrical stimulation and neutral audiovisual stimuli). Mean duration of the anticipation epochs was identical across trial types, ensuring an equal number of measurements (TRs/condition). The specific mean duration was chosen to enhance detection of task-related differences in the blood oxygen level-dependent (BOLD) signal [75]. To mitigate potential confusion and eliminate mnemonic demands, a lower-case ‘c’ or ‘u’ was presented at the lower edge of the display throughout the anticipatory epoch. White-noise visual masks (3.2 s) were presented between trials to minimize persistence of the visual reinforcers in iconic memory. Subjects were periodically prompted (following the visual mask) to rate the intensity of fear/anxiety experienced a few seconds earlier, during the *anticipation* period of the prior trial, using a 1 (*minimal*) to 4 (*maximal*) scale and an MRI-compatible response pad (MRA, Washington, PA). Each condition was rated once per scan (16.7% trials). Skin conductance was continuously acquired throughout.

#### Procedures

Prior to fMRI scanning, participants practiced an abbreviated version of the MTC paradigm without electrical stimulation until staff confirmed understanding. Benign and aversive electrical stimulation levels were individually titrated. *Benign Stimulation*. Participants were asked whether they could “reliably detect” a 20 V stimulus and whether it was “at all unpleasant.” If the participant could not detect the stimulus, the voltage was increased by 4 V and the process repeated. If the participant indicated that the stimulus was unpleasant, the voltage was reduced by 4 V and the process was repeated. The final level chosen served as the benign electrical stimulation during the imaging assessment (*M* = 24.43 V, *SD* = 5.48 V). *Aversive Stimulation*. Participants received a 100 V stimulus and were asked whether it was “as unpleasant as you are willing to tolerate.” If the participant indicated that they were willing to tolerate more intense stimulation, the voltage was increased by 10 V and the process repeated. If the participant indicated that the stimulus was too intense, the voltage was reduced by 5 V and the process repeated. The final level chosen served as the aversive electrical stimulation during the imaging assessment (*M* = 121.33 V, *SD* = 45.14 V). Following each scan, staff verbally re-assessed whether the level of stimulation was sufficiently aversive and re-calibrated as necessary. Stimulation levels were similar to prior work in university samples [42]. The groups did not significantly differ in the chosen intensity of benign or aversive electrical stimulation (**Table 1**).

#### Electrical stimuli

Electrical stimuli (100 ms; 2 ms pulses every 10 ms) were generated using an MRI-compatible constant-voltage stimulator system (STMEPM-MRI; Biopac Systems, Inc., Goleta, CA). Stimuli were delivered using MRI-compatible, disposable carbon electrodes (Biopac) attached to the fourth and fifth digits of the non-dominant hand.

#### Visual stimuli

Visual stimuli (1.8 s) were digitally back-projected (Powerlite Pro G5550, Epson America, Inc., Long Beach, CA) onto a semi-opaque screen mounted at the head-end of the scanner bore and viewed using a mirror mounted on the head-coil. A total of 72 aversive and benign photographs were selected from the International Affective Picture System [for details, see 42].

#### Auditory stimuli

Auditory stimuli (0.80 s) were delivered using an amplifier (PA-1 Whirlwind) with in-line noise-reducing filters and ear buds (S14; Sensimetrics, Gloucester, MA) fitted with noise-reducing ear plugs (Hearing Components, Inc., St. Paul, MN). A total of 72 aversive and benign auditory stimuli were adapted from open-access online sources.

#### Skin conductance data acquisition

Skin conductance was continuously acquired during each scan using a Biopac system (MP-150; Biopac Systems, Inc., Goleta, CA). Skin conductance (250 Hz; 0.05 Hz high-pass) was measured using MRI-compatible disposable electrodes (EL507) attached to the second and third digits of the non-dominant hand.

#### MRI data acquisition

MRI data were acquired using a Siemens Magnetom TIM Trio 3 Tesla scanner (32-channel head-coil). Sagittal T1-weighted anatomical images were acquired using a magnetization prepared rapid acquisition gradient echo sequence (TR = 2,400 ms; TE = 2.01 ms; inversion time = 1,060 ms; flip angle = 8°; sagittal slice thickness = 0.8 mm; in-plane = 0.8 × 0.8 mm; matrix = 300 × 320; field-of-view = 240 × 256). A T2-weighted image was collected co-planar to the T1-weighted image (TR = 3,200 ms; TE = 564 ms; flip angle = 120°). To enhance resolution, a multi-band sequence was used to collect oblique-axial echo planar imaging (EPI) volumes (multiband acceleration = 6; TR = 1,250 ms; TE = 39.4 ms; flip angle = 36.4°; slice thickness = 2.2 mm, number of slices = 60; in-plane resolution = 2.1875 × 2.1875 mm; matrix = 96 × 96). Images were collected in the oblique axial plane (approximately −20° relative to the AC-PC plane) to minimize potential susceptibility artifacts. A total of three 478-volume EPI scans were acquired. The first seven volumes were automatically discarded by the scanner. To enable fieldmap correction, two oblique-axial spin echo (SE) images were collected in each of two opposing phase-encoding directions (rostral-to-caudal and caudal-to-rostral) at the same location and resolution as the functional volumes (i.e., co-planar; TR = 7,220 ms; TE = 73 ms).

#### Skin conductance data pipeline

Skin conductance data were processed using *PsPM* (version 4.0.2) and in-house Matlab (version 9.9.0.1467703) code [76, 77]. Data were regressed to remove pulse and respiration signals and de-spiked using *filloutliers* (150-sample moving-median widow; modified Akima cubic Hermite interpolation). Each scan was then band-pass filtered (0.009–0.333 Hz), median centered, and down-sampled (4 Hz). Participant-specific skin conductance response functions (SCRFs) were estimated by fitting the four parameters of the canonical SCRF [78] to the grand-average reinforcer response using *fmincon* and a cost function that maximized variance explained and penalized negative coefficients.

#### MRI data pipeline

Methods were optimized to minimize spatial-normalization error and other potential sources of noise. Data were visually inspected before and after processing for quality assurance.

#### Anatomical data processing

Methods are similar to those described in other recent reports by our group [42, 43]. T1-weighted images were inhomogeneity corrected using *N4* [79] and denoised using *ANTS* [80]. The brain was then extracted using *BEaST* [81] and brain-extracted and normalized reference brains from *IXI* [82]. Brain-extracted T1 images were normalized to a version of the brain-extracted 1-mm T1-weighted MNI152 (version 6) template [83] modified to remove extracerebral tissue. Normalization was performed using the diffeomorphic approach implemented in *SyN* (version 2.3.4) [80]. T2-weighted images were rigidly co-registered with the corresponding T1 prior to normalization. The brain extraction mask from the T1 was then applied. Tissue priors were unwarped to native space using the inverse of the diffeomorphic transformation [84]. Brain-extracted T1 and T2 images were segmented—using native-space priors generated in *FAST* (version 6.0.4) [85]—to enable T1-EPI co-registration (see below).

#### Fieldmap data processing

SE images and *topup* were used to create fieldmaps. Fieldmaps were converted to radians, median-filtered, and smoothed (2-mm). The average of the distortion-corrected SE images was inhomogeneity corrected using *N4* and masked to remove extracerebral voxels using *3dSkullStrip* (version 19.1.00).

#### Functional data processing

EPI files were de-spiked using *3dDespike*, slice-time corrected to the TR center using *3dTshift*, and motion corrected to the first volume and inhomogeneity corrected using *ANTS* (12-parameter affine). Transformations were saved in ITK-compatible format for subsequent use [86]. The first volume was extracted for EPI-T1 coregistration. The reference EPI volume was simultaneously co-registered with the corresponding T1-weighted image in native space and corrected for geometric distortions using boundary-based registration [85]. This step incorporated the previously created fieldmap, undistorted SE, T1, white matter (WM) image, and masks. The spatial transformations necessary to transform each EPI volume from native space to the reference EPI, from the reference EPI to the T1, and from the T1 to the template were concatenated and applied to the processed EPI data in a single step to minimize incidental spatial blurring. Normalized EPI data were resampled (2 mm^3^) using fifth-order b-splines. Hypothesis testing focused on anatomically defined regions of interest (ROIs), as detailed below. To maximize anatomical resolution, no additional spatial filters were applied, consistent with recent recommendations [87]. By convention, exploratory whole-brain voxelwise analyses employed data that were spatially smoothed (6-mm) using *3DblurInMask*.

### Skin conductance data exclusions and modeling

#### Data exclusions

Six participants were excluded from skin conductance analyses due to inadequate data quality, indicated by either staff observation at the time of data acquisition or non-positive mean responses to noxious stimulation.

#### First-Level modeling

Robust general linear models (GLMs) were used to separate electrodermal signals associated with the anticipatory periods of the MTC paradigm from those evoked by other aspects of the task (e.g., reinforcer delivery). Modeling was performed separately for each participant and scan using *robustfit*. Subject-specific SCRFs were convolved with rectangular regressors time-locked to the presentation of the reinforcers (separately for each trial type), visual masks, and rating prompts. To quantify skin conductance level (SCL) during the anticipation (‘countdown’) epochs, first-level residuals were averaged separately for each participant and trial type.

### fMRI data exclusions and modeling

#### Data exclusions

Participants who responded to <50% of rating prompts—indicating poor task compliance—were excluded from all analyses (*n* = 2). The remaining participants completed >83% of the ratings (**Table 1**). Volume-to-volume displacement (>0.5 mm) was used to assess residual motion artifact. Scans with excessively frequent artifacts (>3 *SD*) were discarded. The remaining participants provided at least 2 scans of usable data.

#### Canonical first-level modeling

Single-participant (‘first-level’) GLMs were used to separate hemodynamic signals associated with the anticipatory periods of the MTC paradigm from those evoked by other aspects of the task. GLMs were implemented in *SPM12* (version 7771) using the default autoregressive model and the temporal band-pass filter set to the hemodynamic response function (HRF) and 128 s [88]. Anticipatory signals were modeled using variable-duration rectangular regressors time-locked to the countdown periods of the Uncertain Threat, Certain Threat, and Uncertain Safety trials; and convolved with a canonical HRF and its temporal derivative. To maximize design efficiency, Certain Safety anticipation—which is psychologically similar to a conventional inter-trial interval—served as the implicit baseline. Periods corresponding to the presentation of the reinforcers (separately for each trial type), visual masks, and rating prompts were simultaneously modeled using the same approach. Consistent with prior work using the MTC paradigm [42, 43], nuisance variates included estimates of volume-to-volume displacement, motion (6 parameters × 3 lags), cerebrospinal fluid (CSF) signal, instantaneous pulse and respiration rates, and ICA-derived nuisance signals (e.g. brain edge, CSF edge, global motion, white matter) [89]. Volumes with excessive volume-to-volume displacement (>0.5 mm) and those during and immediately following reinforcer delivery were censored.

#### Extended amygdala regions of interest (ROIs)

Consistent with prior work by our group, task-related Ce and BST activation was quantified using well-established, anatomically defined ROIs and spatially unsmoothed fMRI data in atlas space [87] (**Fig 1**). The derivation of the Ce ROI is detailed in Tillman et al. (2018). The probabilistic BST ROI was developed by Theiss and colleagues and was thresholded at 25% [90]. It mostly encompasses the supra-commissural BST, given the difficulty of reliably discriminating the borders of regions below the anterior commissure in T1-weighted images [91]. Bilateral ROIs were decimated to the 2-mm resolution of the fMRI data. EAc ROI analyses used standardized regression coefficients extracted and averaged for each combination of task contrast (e.g., Uncertain Threat anticipation vs. Uncertain Safety anticipation), ROI, and participant. *A priori* anatomically defined ROIs provided unbiased estimates of brain-phenotype associations [i.e., statistically unbiased effect size estimates; 48]. Key conclusions remained unchanged when Certain and Uncertain Threat were contrasted with the implicit baseline (Certain Safety anticipation; not reported).

### Analytic strategy

#### Overview

Except where noted otherwise, analyses were performed using *R* (version 4.0.2) [92] and *RStudio* [93]. Descriptive psychometric analyses were performed using *psych* (version 2.2.5) [94]. Some figures were created using *ggpubr* (version 0.4.0) [95] and MRIcron [96]. To facilitate interpretation, standardized effect sizes (Cohen’s *d*) were computed for key group mean differences using *JASP* (version 0.16.3) [97].

The overarching goal of this project was to perform a multimodal assessment of the impact of 24-hour nicotine abstinence, with a major focus on the consequences of acute abstinence for EAc threat reactivity. To that end, hypothesis testing focused on four measurement modalities (‘readouts’): **(a)** questionnaire measures of subjective smoking urges (BQSU) and withdrawal symptoms (WSWS), including anxious withdrawal (WSWS-Anxiety); **(b)** in-scanner ratings of subjective fear and anxiety elicited by threat anticipation; **(c)** concurrent measures of threat-evoked physiological arousal (SCL); and **(d)** and fMRI measures of EAc threat reactivity (Ce and BST ROIs; spatially unsmoothed data).

#### Confirmatory testing

In combination with the breath CO assessment performed at the outset of the neuroimaging session (**Table 1**), the questionnaire measures of smoking urges and withdrawal served as a check on the validity of the abstinence manipulation, while the ratings and SCL measures served as a check on the validity of the threat-anticipation (MTC) paradigm [98, 99]. As a further check, we confirmed that the MTC paradigm activated the canonical threat-anticipation network, including the dorsal amygdala (Ce) and BST [42, 43, 100]. Spatially smoothed data (6-mm kernel) and whole-brain voxelwise (‘second-level’) repeated-measures GLMs (‘random effects’) were used to compare each threat-anticipation condition with its corresponding control condition (e.g., Uncertain Threat vs. Uncertain Safety). Significance was assessed using FDR *q* < .05, whole-brain corrected. As in prior work by our group [42], a minimum conjunction test (logical ‘AND’) was used to identify voxels sensitive to both temporally Certain *and* Uncertain Threat anticipation [101]. Finally, a series of one-sample Student’s *t*-tests was used to confirm that the EAc ROIs—which capitalized on spatially unsmoothed data—showed significant activation during Certain and Uncertain Threat anticipation.

#### Hypothesis testing

A series of independent Student’s *t*-tests were used to confirm the expectation that the 24-Hour Abstinence group would experience elevated smoking urges and withdrawal. Standard mixed-effects GLMs—implemented using *afex* (version 1.1–1)—were used to test whether acute abstinence potentiates subjective (fear/anxiety ratings) and objective psychophysiological (SCL) reactivity to the threat-anticipation paradigm [102]. Significant interactions (e.g., Group × Valence) were decomposed using focal independent or dependent Students *t*-tests. The same approach was used to test the central hypothesis that EAc threat reactivity would be potentiated in the 24-Hour Abstinence group, and to explore the possibility that hyper-reactivity would be more evident during Uncertain Threat anticipation [31]. It merits comment that—because this analysis used contrasts (Uncertain Threat–Uncertain Safety; Certain Threat–Certain Safety)—the main effect of ‘ROI’ is conceptually and statistically equivalent to testing the Region × Valence interaction. On an exploratory basis, we also performed a series of whole-brain voxelwise GLMs to identify potential group differences in activation during Certain and Uncertain Threat anticipation (FDR *q* < .05, whole-brain corrected). Sensitivity analyses indicated that none of the conclusions changed when mean-centered age was included as a nuisance variate (results not reported). Likewise, none of the conclusions drawn from the ROI analyses materially changed when we included mean-centered Ce/BST temporal signal-to-noise (tSNR)—an index of regional signal quality—as a nuisance variate (results not reported).

## Results

### Acute nicotine abstinence increases smoking urges and withdrawal symptoms

As shown in **Table 1** and **S1 Fig**, the 24-Hour Abstinence group reported significantly greater smoking urges (BQSU), general withdrawal symptoms (WSWS), and anxious withdrawal (WSWS Anxiety) just prior to scanning, reinforcing the validity of our abstinence manipulation (*t*s(73)>5.22, *p*s<0.001, Cohen’s *d*s>1.21).

### Acute nicotine abstinence potentiates subjective reactivity to threat

**Fig 2:**
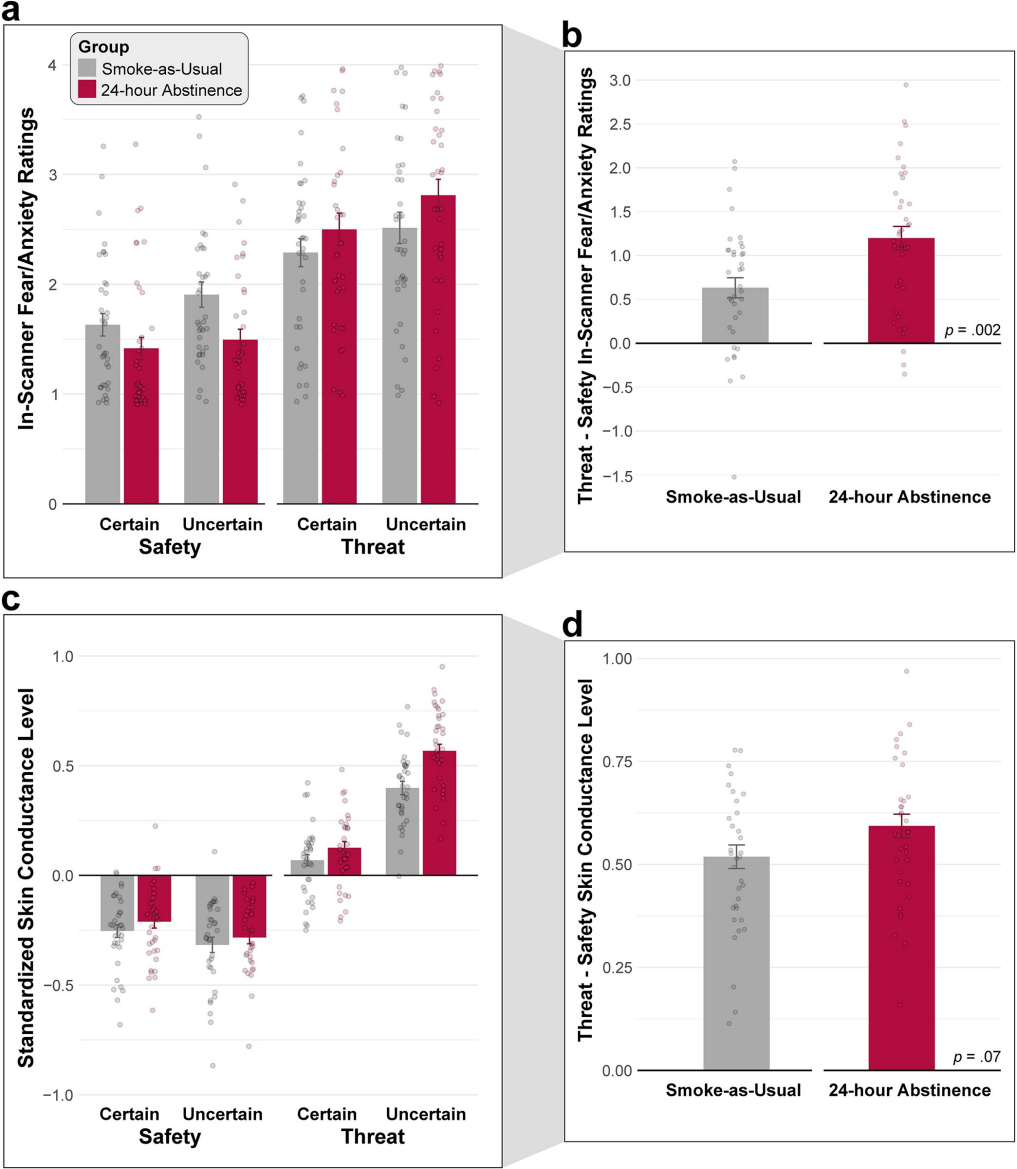
The impact of acute nicotine abstinence on subjective distress and objective physiological arousal elicited by the threat-anticipation paradigm. *Upper panels*. Mean self-reported intensity of fear and anxiety experienced during the anticipation epoch of each condition for the Smoke-as-Usual (*grey*) and 24-hour Abstinence (*red*) groups. Participants were quasi-randomly prompted to rate each condition three times while completing the MTC paradigm. *Lower panels*. Mean SCL during the anticipation epochs of the MTC. (a) Consistent with prior work, distress was significantly elevated during the anticipation of Threat compared to Safety, and during the anticipation of temporally Uncertain compared to Certain reinforcers (*p*s<0.001). (b) The 24-Hour Abstinence group experienced significantly intensified fear and anxiety during the anticipation of Threat compared to Safety (Group × Valence, *p* = 0.002). (c) Psychophysiological arousal was also significantly increased during the anticipation of Threat compared to Safety, reinforcing the validity of the MTC paradigm (*p*<0.001). This increase was particularly evident during the anticipation of temporally Uncertain compared to Certain reinforcers (Valence × Certainty, *p*<0.001). On average, abstinent participants showed significantly greater arousal throughout the MTC paradigm (inclusive of all conditions) compared to those who smoked as usual (*p* = 0.008). (d) Echoing the subjective distress results, the 24-Hour Abstinence group showed a trend toward greater arousal during Threat anticipation (Group × Valence, *p* = 0.07). Bars indicate group means, whiskers indicate SEs, and dots indicate participant-level means.

As shown in **Fig 2A**, fearful and anxious feelings were significantly elevated during the anticipation of Threat compared to Safety, and during the anticipation of temporally Uncertain compared to Certain reinforcers (Valence: *F*(1,73) = 108.73, *p*<0.001; Certainty: *F*(1,73) = 32.53, *p*<0.001).

Notably, the 24-Hour Abstinent group reported significantly more intense fear and anxiety during Threat (vs. Safety) anticipation, compared to the Smoke-as-Usual group (Group × Valence: *F*(1,73) = 10.40, *p* = 0.002, Cohen’s *d* = 0.75; 24-Hour Abstinence, Threat vs. Safety: *t*(37) = 9.05, *p*<0.001; Smoke-as-Usual, Threat vs. Safety: *t*(36) = 5.51, *p*<0.001). The group difference in distress was numerically larger when threat was temporally uncertain, but this difference was not statistically significant (Group × Valence × Certainty, *F*(73) = 2.39, *p* = 0.13). Other effects were not significant, *p*s>0.13. In short, acute nicotine abstinence amplifies threat-evoked distress, and does so similarly across Certain and Uncertain threat contexts (**Fig 2B**).

### Acute nicotine abstinence non-specifically increases physiological arousal

As shown in **Fig 2C**, SCL—an objective psychophysiological index of arousal—was significantly increased during the anticipation of Threat compared to Safety, and this was particularly evident during the anticipation of temporally Uncertain reinforcers (Valence: *F*(1,67) = 746.50, *p*<0.001; Certainty: *F*(1,67) = 101.86, *p*<0.001; Valence × Certainty: *F*(1,67) = 746.50, *p*<0.001; Threat, Uncertain vs. Certain: *t*(68) = 12.72, *p*<0.001; Safety, Uncertain vs. Certain: *t*(68) = -3.89, *p*<0.001). These observations reinforce the validity of the MTC paradigm as an experimental probe of fear and anxiety.

Averaged *across* conditions of the MTC paradigm, abstinent smokers showed significantly greater physiological arousal than those who smoked as usual (*F*(1,67) = 7.56, *p* = 0.008, Cohen’s *d* = 0.66). Echoing the results for subjective distress, the 24-Hour Abstinence group also showed a trend toward greater arousal during Threat anticipation (Group × Valence: *F*(1,67) = 2.59, *p* = 0.071, Cohen’s *d* = 0.44; 24-Hour Abstinence, Threat vs. Safety: *t*(34) = 20.52, *p*<0.001; Smoke-as-Usual, Threat vs. Safety: *t*(33) = 18.13, *p*<0.001). The group difference in arousal was numerically larger when threat was temporally uncertain, but this difference was not statistically significant (Group × Valence × Certainty, *F*(67) = 2.59, *p* = 0.11). Other effects were not significant, *p*s>0.10. Collectively, these observations show that acute nicotine abstinence non-specifically increases physiological arousal during periods of threat and safety anticipation (red vs. gray bars, **Fig 2C**), and provide weak convergent evidence of potentiated reactivity to threat (**Fig 2D**).

### The MTC paradigm recruits the canonical threat-anticipation network, including the EAc

**Fig 3:**
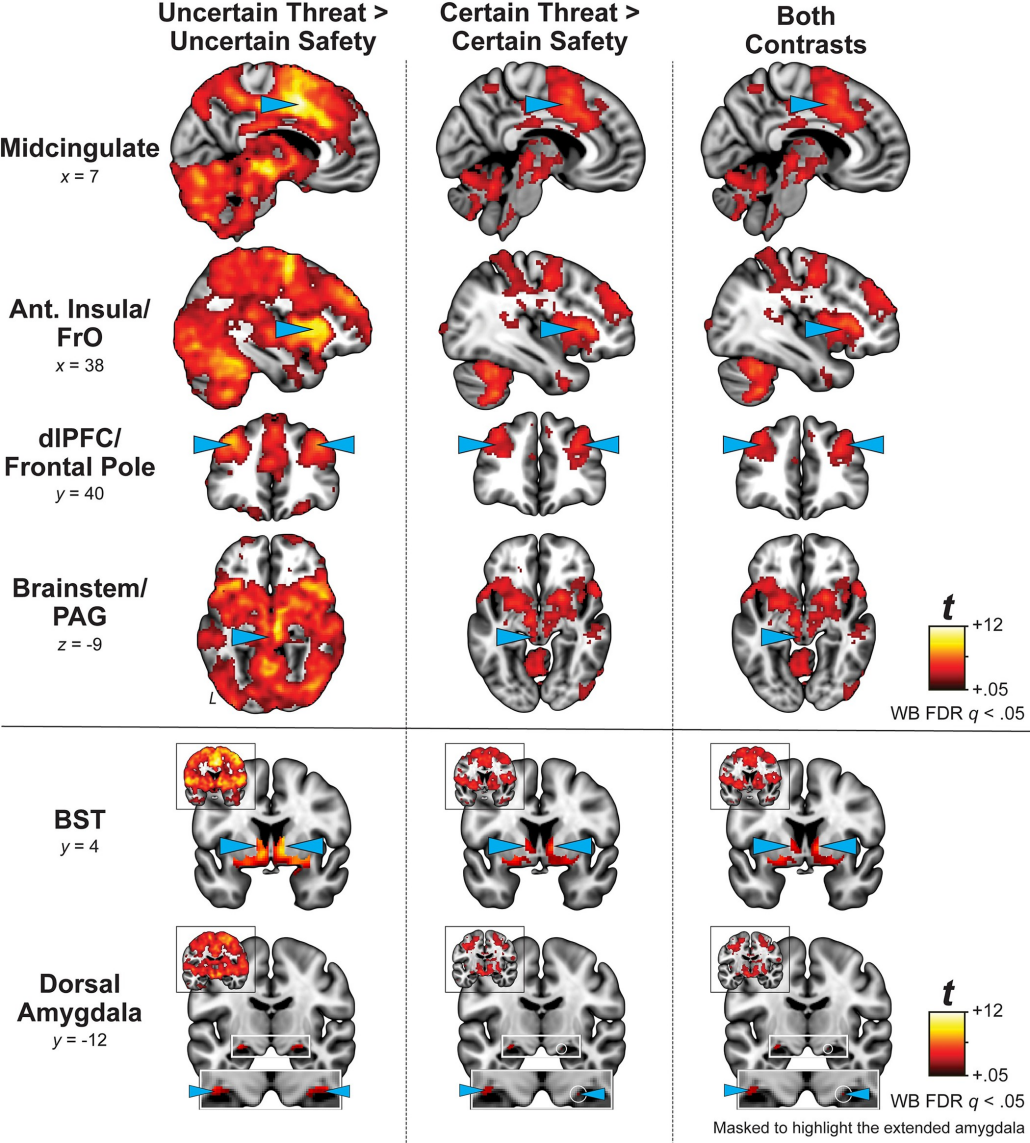
Uncertain and Certain Threat anticipation recruit a common cortico-subcortical network. Key regions (*cyan arrowheads*) show significantly increased activation during the anticipation of both Uncertain Threat (*left column*) and Certain Threat (*middle column*) compared to their respective control conditions (FDR *q*<0.05, whole-brain corrected). Both threat conditions recruited a common neural circuit—including the BST and dorsal amygdala (Ce)—replicating prior work in university students [42]. *Right column* depicts the voxelwise overlap (Logical AND of the thresholded maps depicted in the left and middle columns). BST and dorsal amygdala images are masked to highlight the extended amygdala. Coronal insets show the corresponding statistical parametric maps without the extended amygdala mask. Abbreviations—Ant., anterior; dlPFC, Dorsolateral Prefrontal Cortex; FrO, Frontal Operculum; BST, Bed Nucleus of the Stria Terminalis; FDR, False discovery rate; PAG, Periaqueductal Gray; WB, whole-brain corrected.

We used a series of whole-brain voxelwise GLMs to determine whether that the MTC paradigm had the intended consequences on brain function. As expected, Uncertain Threat anticipation was associated with significant activation across a widely distributed network of regions previously implicated in the expression and regulation of human fear and anxiety [100], including the midcingulate cortex (MCC); anterior insula (AI) extending into the frontal operculum (FrO); dorsolateral prefrontal cortex (dlPFC) extending to the frontal pole (FP); brainstem encompassing the periaqueductal grey (PAG); basal forebrain, in the region of the BST; and dorsal amygdala (Uncertain Threat > Uncertain Safety; FDR *q* < .05, whole-brain corrected; **Fig 3** and **S3 and S4 Tables**).

We used a parallel approach to identify regions recruited during the anticipation of temporally Certain Threat (Certain Threat > Certain Safety; FDR *q* < .05, whole-brain corrected). As shown in the middle column of **Fig 3**, results were notably similar to those evident during Uncertain Threat anticipation (**S5, S6 Tables**). In fact, as shown in the right column of **Fig 3**, a minimum conjunction analysis [Logical ‘AND;’ 101] revealed voxelwise co-localization in every key cortical and subcortical region, including the BST and dorsal amygdala in the region of the Ce (**S7 Table**). These observations replicate prior work in university samples, confirm that the MTC paradigm robustly engages the canonical threat anticipation network—including the EAc—and set the stage for testing the impact of acute nicotine abstinence on EAc threat reactivity [42, 43].

### Acute nicotine abstinence had a negligible impact on EAc threat reactivity

**Fig 4:**
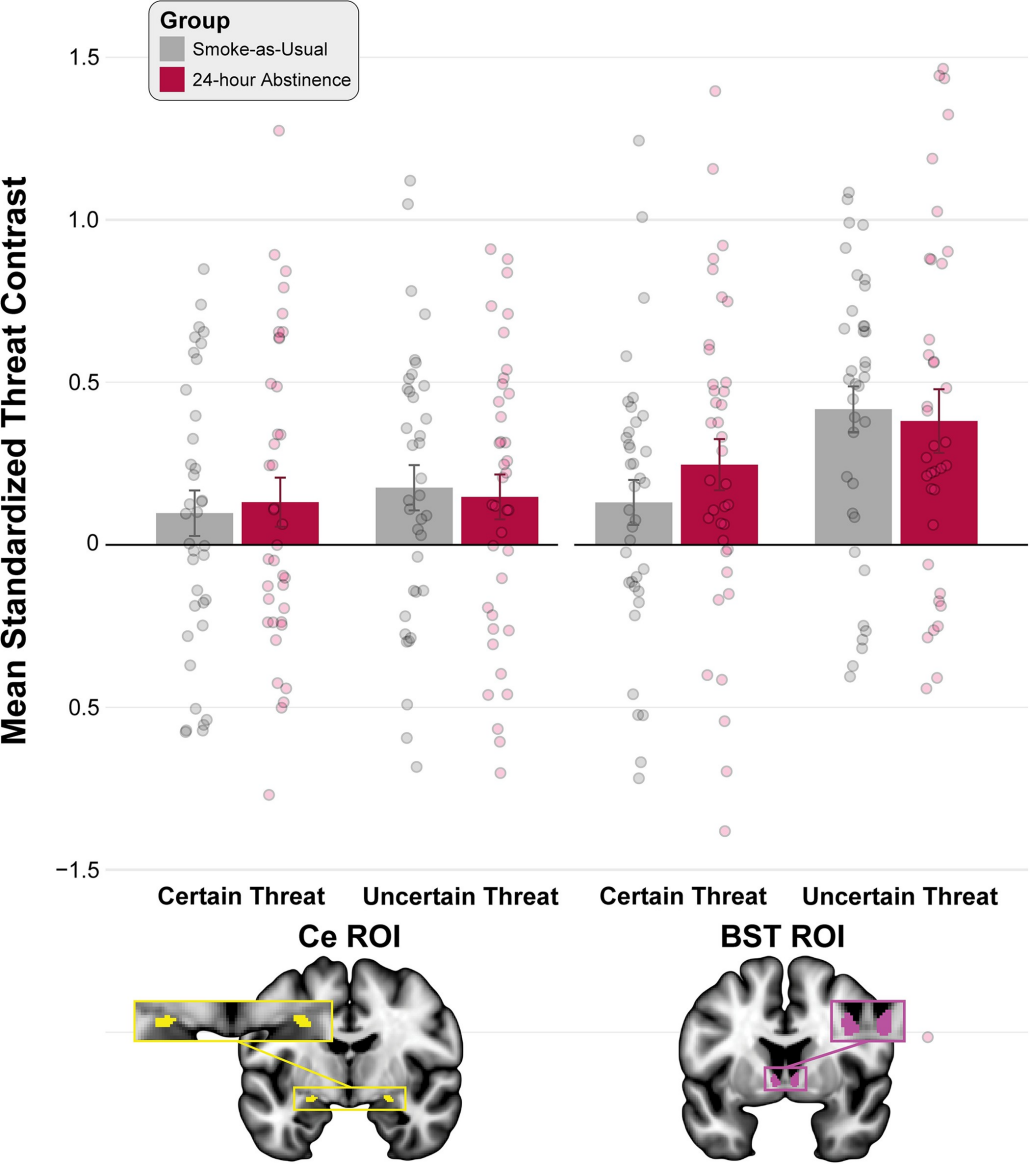
The impact of threat anticipation and acute nicotine abstinence on the EAc. Figure depicts mean contrast coefficients (Threat—Safety) during the anticipation of temporally Certain or Uncertain Threat—relative to their respective control conditions—in the anatomically defined Ce (*yellow*) and BST (*magenta*) ROIs for the Smoke-as-Usual (*grey*) and 24-hour Abstinence (*red*) groups. The BST was more sensitive to threat—irrespective of temporal certainty—compared to the Ce (*p* = 0.003). On average, the two divisions of the EAc showed greater activation during Uncertain Threat anticipation (*p* = 0.02). Interpretation of these effects is tempered by a trend-level Region × Threat Certainty interaction (*p* = 0.10). The Ce showed similarly heightened activation during Certain and Uncertain Threat anticipation (*p* = 0.52), whereas the BST showed a clear preference for Uncertain Threat (*p* = 0.007). Other effects were nonsignificant (*p*s>0.33), indicating that acute nicotine abstinence had a negligible impact on EAC threat reactivity. Bars indicate group means, whiskers indicate SEs, and dots indicate participant-level means. Abbreviations—BST, bed nucleus of the stria terminalis; Ce, central nucleus of the amygdala; ROI, region of interest.

**Fig 5:**
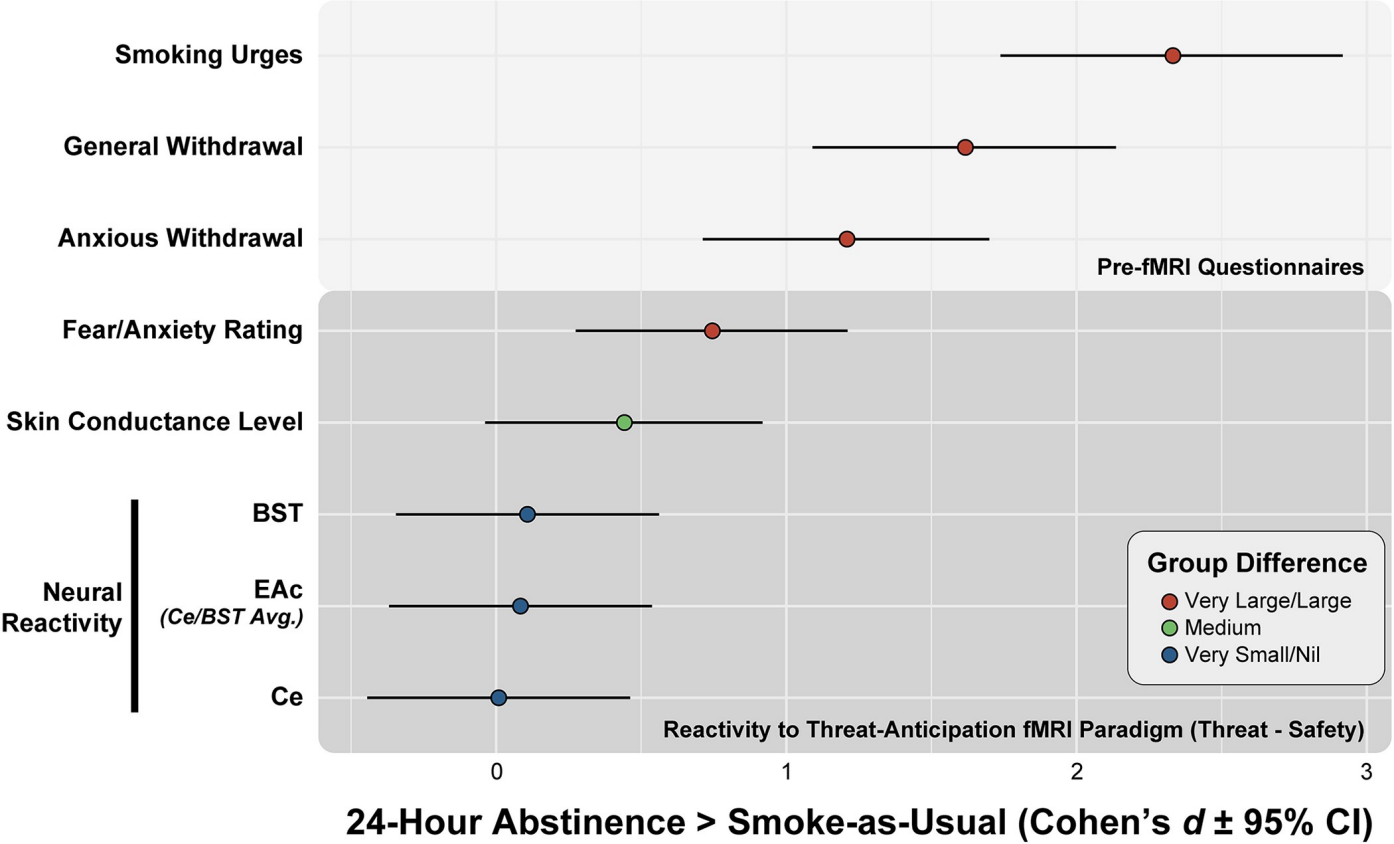
Forest plot summarizing the consequences of acute nicotine abstinence across key outcomes. To facilitate comparison, results are depicted as standardized group mean differences (*dots*; Cohen’s *d*). Whiskers indicate the precision of the standardized differences (95% confidence interval). *Upper panel* (*light grey*) shows group differences for subjective feelings of smoking urgency and withdrawal, both assessed just prior to scanning. *Lower panel* (*dark grey*) shows standardized group differences for the threat-reactivity (Threat minus Safety) measures acquired during fMRI scanning, including threat-evoked changes in subjective fear and anxiety, objective physiological arousal (SCL), and EAc activation. While all of the standardized group differences were in the expected positive direction (24-Hour Abstinence > Smoke-as-Usual), there were marked differences in magnitude across outcomes. Nicotine abstinence exerted a large to very large (*red*) impact on all of the self-report measures of subjective experience (*d*s = 0.75–2.33). In contrast, nicotine abstinence exerted a more moderate influence on threat-elicited arousal (*green*; *d* = 0.44) and a very small to nil impact on the fMRI measures of EAc threat reactivity (*blue*; *d*s = 0.01–0.11). Effect sizes are interpreted with reference to widely used benchmarks (*Large*, *d* = 0.80; *Medium*, *d* = 0.50; *Small*, *d* = 0.20) [138]. Abbreviations—BST, bed nucleus of the stria terminalis; Ce, central nucleus of the amygdala; CI, confidence interval; EAc, central extended amygdala; fMRI, functional magnetic resonance imaging.

We leveraged anatomically defined Ce and BST ROIs and spatially unsmoothed fMRI data to rigorously test the prediction that acute nicotine abstinence amplifies EAc reactivity to threat, and to explore the possibility that amplification would be more evident during Uncertain Threat anticipation (**Fig 1**). As a precursor to hypothesis testing, we used a series of *t*-tests to confirm that the Ce and BST ROIs exhibit significant activation during the anticipation of Certain and Uncertain Threat, relative to their respective control conditions (**Fig 4**). Consistent with the voxelwise results (**Fig 3**), both ROIs were significantly recruited by both kinds of Threat, *t*s(75)>2.22, *p*s<0.03 (**S8 Table**). We then used a standard mixed-effects GLM for hypothesis testing. As shown in **Fig 4**, results revealed the BST was significantly more sensitive to threat anticipation—irrespective of threat certainty—compared to the Ce (Region: *F*(1,73) = 9.27, *p* = 0.003). On average, the EAc showed significantly greater activation during the anticipation of temporally Uncertain Threat (Threat Certainty: *F*(1,73) = 5.39, *p* = 0.02). Interpretation of these effects is somewhat tempered by a trend-level Region × Threat Certainty interaction (*F*(73) = 2.80, *p* = 0.10). Focal comparisons indicated that that the Ce showed an indiscriminate pattern of reactivity, with statistically indistinguishable levels of heightened activation during the anticipation of Certain and Uncertain Threat (*t*(74) = 0.65, *p* = 0.52). In contrast, the BST showed a more pronounced preference for responding to Uncertain Threat anticipation (*t*(74) = 2.80, *p* = 0.007). No other GLM effects were significant (*p*s>0.33), indicating that acute nicotine abstinence had a negligible impact on EAc threat reactivity, contrary to prediction. Although the 24-Hour Abstinence group did show numerically greater EAc activation during threat anticipation, as hypothesized, the standardized group differences were non-significant (*t*s(73)<0.03 *p*s>0.64) and very small to nil (Cohen’s *d*s: Ce = 0.009, BST = 0.11, EAc Average = 0.08; **Fig 5**). Likewise, exploratory whole-brain voxelwise analyses did not uncover any regions showing significant nicotine abstinence effects for either Certain or Uncertain Threat (FDR *q*<0.05, whole-brain corrected), perhaps reflecting the highly distributed neural substrates of subjective distress [103, 104].

### Different modalities provide unique perspectives on the impact of acute nicotine abstinence

The overarching goal of this project was to develop a more complete understanding of the psychobiological impact of acute nicotine abstinence and, to that end, we acquired a multimodal battery of subjective (questionnaires and ratings) and objective (skin conductance and fMRI) measures. A natural question concerns the degree to which those ‘readouts’ reflect a single or small number of latent states (e.g., ‘abstinence’ or ‘anxiety’). Do subjective reports of threat-elicited distress covary with objective signs of physiological arousal, for instance? Does BST threat reactivity covary with individual differences in the subjective intensity of smoking urges? To address these questions and inform the interpretation of our primary hypothesis tests, we computed Spearman correlations across all of the measures acquired at the neuroimaging session, both for the entire sample and for the 24-Hour Abstinence group (**S9 and S10 Tables**). While we detected numerous nominally significant associations, on balance, the four measurement modalities showed a relatively weak and inconsistent pattern of covariance, in broad accord with work underscoring the modest coherence (‘concordance’ or ‘synchrony’) of emotion readouts [105, 106]. In sum, each of the four measurement modalities acquired here provides a distinct and relatively independent perspective on the consequences of acute nicotine abstinence.

## Discussion

Tobacco smoking imposes a staggering burden on global public health [1, 5]. Relapse is common and existing cessation aids are far from curative, underscoring the urgency of developing a fuller understanding of the consequences of acute nicotine abstinence and withdrawal [2, 5, 107, 108]. Converging lines of clinical and experience sampling research highlight the importance of anxious withdrawal symptoms and negative reinforcement mechanisms for maintaining tobacco use in humans, but the underlying neurobiology has remained elusive, impeding therapeutics development [5, 14, 15]. Mechanistic work in animals suggests that anxious withdrawal reflects neuroplastic alterations in EAc neurotransmission that promote exaggerated stressor reactivity in nicotine-dependent rats and mice, but the translational relevance of these discoveries to the complexities of the human brain and human emotion remain unclear [5, 9, 10]. Here we leveraged a novel combination of subjective, psychophysiological, and neurobiological approaches to understand the impact of 24-hour nicotine abstinence in daily tobacco smokers (**Fig 1**). Results demonstrated that the abstinence manipulation worked as intended, potently increasing smoking urges and subjective symptoms of withdrawal, including anxious mood (**S1 Fig**). The threat-anticipation paradigm also had the expected consequences, amplifying subjective and somatic reactivity to threat, and recruiting the canonical threat-anticipation network, including the BST and dorsal amygdala (Ce) (**Figs 2–4**).

Acute abstinence had robust consequences for subjective responses to the threat-anticipation paradigm, amplifying the intensity of threat-evoked fear and anxiety (**Fig 2B**). A similar trend (*p* = 0.07) was evident for threat-evoked arousal (**Fig 2D**). Acute abstinence was also associated with a tonic increase in arousal across the experimental threat and safety contexts (**Fig 2C**). Across these two measures, the magnitude of abstinence-induced increases in threat reactivity was similar across certain and uncertain contexts, suggesting less specificity than that observed in some prior psychophysiological studies [31]. Although the 24-Hour Abstinence group did show numerically greater EAc activation in the threat contexts, as hypothesized, standardized group differences were negligible and whole-brain analyses did not uncover any other regions showing significant abstinence effects (**Fig 4**). Across ‘readouts’, acute abstinence showed a large to very large impact on all of the self-report measures of subjective experience, including tonic measures of smoking urges and withdrawal, and reactive measures of threat-elicited distress. In contrast, abstinence exerted a moderate influence on threat-elicited arousal, and a very small to nil impact on fMRI measures of EAc threat reactivity (**Fig 5**). Follow-up analyses revealed weak coherence across measures, suggesting that they provide comparatively independent perspectives on the consequences of acute nicotine abstinence (**S9 and S10 Tables**). Collectively, these new observations provide a framework for conceptualizing the impact of acute nicotine abstinence and anxious withdrawal, with implications for basic, translational, and clinical science.

Clinical, observational (e.g. EMA), experimental, and animal behavioral data suggest that withdrawal-related negative affect and heightened stressor reactivity are key triggers of lapses during nicotine cessation attempts [10, 14, 16, 24, 28–30]. The present results reinforce this hypothesis. Leveraging a randomized study design, well-matched groups, and a potent experimental stressor, we observed robust increases in threat-evoked fear and anxiety (**Figs 1, 2 and 5**; **Table 1**). Our approach—which leveraged near-real-time ratings collected on a random subset of trials—circumvents the recall biases that can influence retrospective (end-of-task or end-of-session) assessments. Skin conductance evinced a parallel trend, tempering potential concerns centered on demand characteristics. Taken together, this body of work reinforces the significance of withdrawal-related anxiety and heightened stressor reactivity, and points to the need to develop cessation interventions that more effectively dampen stressor reactivity [21, 109–111]. Psychosocial treatments aimed at cultivating stronger emotion regulation skills or weakening the link between distress and smoking may be especially useful [2].

Work in rodents suggests that the pervasive increases in anxiety (tonic) and heightened surges of stressor-evoked distress (reactive) that often accompany periods of abstinence in nicotine-dependent humans reflect hyper-reactivity to threat in the EAc [5, 9, 10]. Yet our unbiased ROI results show that the impact of 24-hour nicotine abstinence on EAc threat reactivity is negligible and largely unrelated to the subjective symptoms—the elevated smoking urges, withdrawal symptoms, and stressor-evoked distress—thought to maintain addiction in human smokers (**Figs 4, 5** and **S9 and S10 Tables**).

How should we interpret this apparent failure of translation? One possibility is that the mechanistic insights gleaned from animal models of nicotine dependence are fundamentally correct, but conventional fMRI approaches—which rely on bulk changes in regional blood oxygenation—are insufficiently sensitive to underlying alterations in EAc molecular signaling and neuronal function [112]. Or, it might be that the threat-anticipation paradigm used here and in prior human psychophysiology studies is suboptimal (“wrong” human assay). Most of the anxiety assays used in rodent models of addiction—conditioned-place avoidance, elevated-plus maze, open field, shock-probe burying—focus on instrumental defensive behaviors emitted over extended assessment periods (5–15 minutes) [35, 36, 113–115]. In contrast, the Maryland Threat Countdown and other popular threat-anticipation assays (e.g., NPU, Pavlovian threat conditioning) are structured as randomly intermixed, relatively brief (5–90 s) periods of safety and threat [64, 116]. To the extent that nicotine abstinence causes persistent, comparatively sluggish alterations in EAc threat reactivity—as one might expect based on the temporal dynamics of stress- and deprivation-induced changes in EAc neurotransmission [e.g., 35, 117]—such ‘event-related’ repeated-measures designs may be suboptimal for detecting neural signs of threat hyper-reactivity. This speculation is consistent with the robust non-specific increase in somatic arousal that we observed in the 24-hour Abstinence group (**Fig 2C**). It is also consistent with the results of the only startle study that employed a between-subjects design. In a seminal 2010 report, Hogle and colleagues demonstrated that 24-hour nicotine abstinence magnified startle reactivity during a ~10-min block of temporally uncertain threat (shock), but was without effect in an independent sample of deprived smokers exposed to an intermixed block of certain-threat and certain-safety cues [31]. Although conventional fMRI sequences cannot quantify minutes-long changes in neural activity, recently established multiband perfusion (‘arterial spin labeling’) MRI sequences can do so with adequate anatomical resolution (<3-mm^3^). Paired with a slow block-related design (e.g., 5-min baseline, 5-min uncertain threat, 5-min recovery), this approach would afford the opportunity to examine abstinence-induced alterations in tonic, reactive, and persistent (‘spill-over’) EAc activity. It would also afford the opportunity to explore stressors that more closely resemble those evident in real-world settings (e.g., social stressors). While we cannot rule out the possibility that the structure of our threat-anticipation paradigm is suboptimal for gauging the impact of nicotine abstinence on EAc function, the medium-to-large effects evident for stressor-potentiated distress and arousal (Valence and Group × Valence effects), and the robust impact on EAc activation across the two groups, run counter to this argument (**Figs 2 and 3**).

Another possibility is that our results represent a genuine translation failure; that the neurobiological mechanisms identified in rodents are sound, but that shock-probe burying and other instrumental defensive behaviors are a suboptimal means of modeling the subjective feelings of anxiety and distress that are central to withdrawal and relapse among abstinent human smokers (“wrong” animal assay). We are not the first commentators to raise this concern [52, 57, 106, 118]. It is broadly consistent with both the comparatively weak coherence that we observed across readouts and with the failure of several novel therapeutic compounds—which emerged from rodent models of anxiety and addiction—to show efficacy in human clinical and experimental-therapeutics trials [16, 45, 119]. For example, corticotropin-releasing hormone (receptor 1) antagonists have been shown to *exacerbate* subjective anxiety elicited by a social stressor (Trier Social Stress Task) and *potentiate* startle reactivity to certain-and-imminent shock delivery, despite evidence of *reduced* amygdala glucose metabolism and *blunted* amygdala reactivity to emotional faces [44, 120, 121]. On balance, these considerations underscore the need to develop coordinated cross-species models of abstinence-induced anxiety. An optimal translational model of withdrawal-induced anxiety would rely on broadly similar procedures across species (e.g., 5–10 min exposure to temporally uncertain shock), show similar behavioral signals across species (e.g., deprivation-induced startle potentiation), show evidence of behavioral relevance across species (e.g., associations with consumption, reinstatement, or lapses), show evidence of experiential relevance in humans (e.g., heightened symptoms of anxious withdrawal and craving), and be amenable to functional neuroimaging in both species (e.g., perfusion fMRI). Demonstrating consistent effects across rodent strains or species would further enhance confidence in translational relevance [122, 123]. Preclinical studies of anxiety in nonhuman primates underscore the feasibility and added-value of this approach [34, 124, 125].

A final possibility is that we simply lack the power to detect modest abstinence-induced changes in EAc threat reactivity. As detailed in the Method, our study was powered to detect medium-to-large effects (Cohen’s *d*>0.66). Inspection of the 95% confidence intervals shown in **Fig 5** raise the possibility that the true impact of acute nicotine abstinence on EAc threat reactivity (as indexed using the present approach) could plausibly be as large as *d* = .50 (‘medium’ effect). Reliably detecting an effect of this magnitude would require ~120 usable datasets. Even under this optimistic scenario, the distribution of EAc threat reactivity would massively (80.3%) overlap across the groups [126]. Put another way, with a medium-sized effect there is only a 63.8% chance that an individual picked at random from the 24-hour Abstinent group would show greater EAc threat reactivity than one picked at random from the Smoke-as-Usual group [126]. These statistical considerations make it unlikely that EAc threat reactivity—at least as indexed using the present neuroimaging approach—could be used as a biomarker of acute nicotine abstinence [127]. It lacks the sensitivity and specificity necessary for practical applications [128].

While not the primary aim of the present project, our neuroimaging findings also shed light on the neurobiology of fear and anxiety. Since the time of Freud, the distinction between certain (‘fear’) and uncertain (‘anxiety’) danger has been a key feature of neuropsychiatric models of emotion—including the National Institute of Mental Health’s influential Research Domain Criteria (RDoC) framework—but the architecture of the underlying neural systems has remained contentious [34, 51–57]. Our whole-brain voxelwise results demonstrate that uncertain-threat anticipation recruits a distributed network of fronto-cortical (MCC, AI/FrO, and dlPFC/FP) and subcortical (PAG, BST, and dorsal amygdala) regions (**Fig 3**). Analyses focused on the anticipation of temporally certain threat revealed a similar pattern, with voxels sensitive to both kinds of threat evident across these key regions (**Fig 3**). These observations dovetail with recent meta-analyses of the neuroimaging literature, replicate prior evidence of anatomical co-location in university students, and reinforce the conclusion that ‘fear’ and ‘anxiety’ reflect a shared set of neural building blocks [42, 129, 130].

Our results also have implications for on-going debates about the functional architecture of the EAc [34]. Inspired by an earlier-generation of lesion studies in rodents [131], it is widely believed that these regions are functionally dissociable, with the amygdala mediating phasic responses to clear-and-immediate danger (‘fear’) and the BST mediating sustained responses to uncertain-or-remote danger (‘anxiety’) [52, 132–134]. This hypothesized double dissociation has even been enshrined in the RDoC framework [56, 135, 136]. Leveraging the enhanced resolution afforded by a best-practices fMRI pipeline and spatially unsmoothed data, our unbiased ROI results demonstrate that the Ce and the BST both show significant engagement across threat contexts, consistent with our voxelwise observations (**Figs 3 and 4; S8 Table**). On average, the EAc showed significantly greater activation during the anticipation of temporally uncertain threat, paralleling the concurrent measures of subjective distress and objective arousal (**Figs 2 and 4**). While the Region × Threat Certainty interaction was only marginally significant (*p* = 0.10), focal comparisons indicated that that the Ce showed an indiscriminate pattern of reactivity, with statistically indistinguishable levels of heightened activation during the anticipation of certain and uncertain threat. In contrast, the BST showed preferential activation during the anticipation of uncertain threat, consistent with a recent large-scale (*n* = 109) study that employed anatomical ROIs [137]. These observations are broadly consistent with the influential model of Davis and colleagues—which suggests that the Ce is involved in responding to both kinds of threat—but run counter to the popular double dissociation model (Ce: Certain >> Uncertain ≈ 0; BST: 0 ≈ Certain << Uncertain) [51]. While our understanding remains far from complete, this body of observations underscores the need to reformulate RDoC and other models of fear and anxiety that imply a strict segregation of certain and uncertain threat processing in the EAc.

Tobacco smoking is a leading preventable cause of morbidity and mortality, and converging lines of evidence underscore the relevance of abstinence-induced anxiety and negative reinforcement mechanisms to the maintenance and treatment of nicotine use. Our experimental results reinforce and extend claims—made largely made on the basis of observational data—that acute nicotine abstinence triggers persistent increases in anxious mood and potentiates subjective stressor reactivity. Our neuroimaging results demonstrate that the impact of acute abstinence on EAc threat reactivity is negligible in humans, raising questions about the translational relevance of popular animal and human experimental models of addiction. A key challenge for the future will be to establish coordinated cross-species models of anxiety and addiction (bi-directional translation). A comprehensive multidimensional approach, well-established threat-anticipation task, and best-practice approaches to data acquisition, processing, and analysis enhance confidence in the robustness and translational relevance of our results. These observations provide a novel framework for conceptualizing anxious withdrawal symptoms and negative reinforcement mechanisms and for accelerating the development of more effective treatment strategies for nicotine dependence.

## Supporting information

S1 Fig The impact of acute nicotine abstinence on mean self-reported **(a)** smoking urges, **(b)** general withdrawal, and **(c)** anxious withdrawal for each group at the neuroimaging session, just prior to scanning. Bars indicate group means, whiskers indicate SEs, and dots indicate individual participants.
(DOCX)

S1 Table Sample demographics, educational attainment. (DOCX)

S2 Table Sample demographics, income. (DOCX)

S3 Table Descriptive statistics for clusters and local extrema showing greater activity during the anticipation of Uncertain Threat compared to Uncertain Safety (FDR *q* < .05, whole-brain corrected). (DOCX)

S4 Table Descriptive statistics for clusters and local extrema showing greater activity during the anticipation of Uncertain Safety compared to Uncertain Threat (FDR *q* < .05, whole-brain corrected). (DOCX)

S5 Table Descriptive statistics for clusters and local extrema showing greater activity during the anticipation of Certain Threat compared to Certain Safety (FDR *q* < .05, whole-brain corrected). (DOCX)

S6 Table Descriptive statistics for clusters and local extrema showing greater activity during the anticipation of Certain Safety compared to Certain Threat (FDR *q* < .05, whole-brain corrected). (DOCX)

S7 Table Descriptive statistics for clusters and local extrema showing greater activity during the anticipation of Uncertain *and* Certain Threat (minimum conjunction of FDR-thresholded maps). (DOCX)

S8 Table One-sample Student’s *t*-tests for central extended amygdala regions of interest (spatially unsmoothed data). (DOCX)

S9 Table Spearman correlations among key outcome measures, all participants. (DOCX)

S10 Table Spearman correlations among key outcome measures, nicotine-abstinent participants. (DOCX)
