# Supplementary material for: Acute nicotine abstinence amplifies subjective withdrawal symptoms and threat-evoked fear and anxiety, but not extended amygdala reactivity
Source: PLoS One. 2023 Jul 20;18(7):e0288544. doi: 10.1371/journal.pone.0288544 (PMC10358993; doi:10.1371/journal.pone.0288544)
Supplement: S1 Table — (DOCX) [file pone.0288544.s002.docx]

**Acute nicotine abstinence amplifies subjective withdrawal symptoms and threat-evoked fear and anxiety, but not extended amygdala reactivity**

Hyung Cho Kim^1,2^

Claire M. Kaplan^4^

Samiha Islam^5^

Allegra S. Anderson^6^

Megan E. Piper^7^

Daniel E. Bradford^8^

John J. Curtin^9^

Kathryn A. DeYoung^1^

Jason F. Smith^1^

Andrew S. Fox^10,11^

Alexander J. Shackman^1,2,3^

^1^Department of Psychology, University of Maryland, College Park, Maryland, United States of America

^2^Neuroscience and Cognitive Science Program, University of Maryland, College Park, Maryland, United States of America

^3^Maryland Neuroimaging Center, University of Maryland, College Park, Maryland, United States of America

^4^Department of Psychiatry and Behavioral Sciences, School of Medicine, Johns Hopkins University, Baltimore, Maryland, United States of America

^5^Department of Psychology, University of Pennsylvania, Philadelphia, Pennsylvania, United States of America

^6^Department of Psychological Sciences, Vanderbilt University, Nashville, Tennessee, United States of America

^7^Center for Tobacco Research and Intervention and Department of Medicine, School of Medicine and Public Health, University of Wisconsin—Madison, Madison, Wisconsin, United States of America

^8^School of Psychological Sciences, Oregon State University, Corvallis, Oregon, United States of America

^9^Department of Psychology, University of Wisconsin—Madison, Madison, Wisconsin, United States of America

^10^Department of Psychology, University of California, Davis, California, United States of America

^11^California National Primate Research Center, University of California, Davis, California, United States of America

Corresponding author(s)

E-mail: [hkim1230@umd.edu](mailto:hkim1230@umd.edu) (HCK), E-mail: [shackman@umd.edu](mailto:shackman@umd.edu) (AJS)

**Supplementary Table S1. Sample demographics, educational attainment.**

| **Highest level of education completed** | **%** |
| --- | --- |
| High school, no degree | 1.3 |
| High school degree or equivalent | 20.0 |
| Some college, no degree | 40.0 |
| Associate degree | 9.3 |
| Bachelor degree | 18.7 |
| Some graduate, no degree | 4.0 |
| Graduate or professional degree | 6.7 |
