# Supplementary material for: Acute nicotine abstinence amplifies subjective withdrawal symptoms and threat-evoked fear and anxiety, but not extended amygdala reactivity
Source: PLoS One. 2023 Jul 20;18(7):e0288544. doi: 10.1371/journal.pone.0288544 (PMC10358993; doi:10.1371/journal.pone.0288544)
Supplement: S3 Table — (DOCX) [file pone.0288544.s004.docx]

**Acute nicotine abstinence amplifies subjective withdrawal symptoms and threat-evoked fear and anxiety, but not extended amygdala reactivity**

Hyung Cho Kim^1,2^

Claire M. Kaplan^4^

Samiha Islam^5^

Allegra S. Anderson^6^

Megan E. Piper^7^

Daniel E. Bradford^8^

John J. Curtin^9^

Kathryn A. DeYoung^1^

Jason F. Smith^1^

Andrew S. Fox^10,11^

Alexander J. Shackman^1,2,3^

^1^Department of Psychology, University of Maryland, College Park, Maryland, United States of America

^2^Neuroscience and Cognitive Science Program, University of Maryland, College Park, Maryland, United States of America

^3^Maryland Neuroimaging Center, University of Maryland, College Park, Maryland, United States of America

^4^Department of Psychiatry and Behavioral Sciences, School of Medicine, Johns Hopkins University, Baltimore, Maryland, United States of America

^5^Department of Psychology, University of Pennsylvania, Philadelphia, Pennsylvania, United States of America

^6^Department of Psychological Sciences, Vanderbilt University, Nashville, Tennessee, United States of America

^7^Center for Tobacco Research and Intervention and Department of Medicine, School of Medicine and Public Health, University of Wisconsin—Madison, Madison, Wisconsin, United States of America

^8^School of Psychological Sciences, Oregon State University, Corvallis, Oregon, United States of America

^9^Department of Psychology, University of Wisconsin—Madison, Madison, Wisconsin, United States of America

^10^Department of Psychology, University of California, Davis, California, United States of America

^11^California National Primate Research Center, University of California, Davis, California, United States of America

Corresponding author(s)

E-mail: [hkim1230@umd.edu](mailto:hkim1230@umd.edu) (HCK), E-mail: [shackman@umd.edu](mailto:shackman@umd.edu) (AJS)

**Supplementary Table S3. Descriptive statistics for clusters and local extrema showing greater activity during the anticipation of Uncertain Threat compared to Uncertain Safety (FDR *q*<.05, whole-brain corrected).**

| **mm^3^** | **Label** | ***t*** | ***x*** | ***y*** | ***z*** |
| --- | --- | --- | --- | --- | --- |
| 1,192,496 | R Frontal Pole | 7.78 | 36 | 50 | 28 |
|  | L Frontal Pole | 8.80 | -32 | 42 | 36 |
|  | L Inferior Frontal Gyrus, pars triangularis | 2.13 | -52 | 30 | 0 |
|  | L Paracingulate/Cingulate Gyri | 8.72 | -8 | 30 | 28 |
|  | L Insular Cortex | 8.90 | -30 | 24 | 6 |
|  | R Subcallosal/Frontal Orbital Cortices | 2.80 | 12 | 24 | -18 |
|  | L Frontal Orbital Cortex | 8.16 | -36 | 22 | -8 |
|  | R Frontal Orbital/Insular Cortices | 8.42 | 36 | 20 | -10 |
|  | R Frontal Operculum Cortex | 9.80 | 36 | 20 | 8 |
|  | L Inferior Frontal Gyrus, pars opercularis | 4.53 | -52 | 18 | 26 |
|  | L Temporal Pole | 4.23 | -46 | 18 | -22 |
|  | L Frontal Operculum Cortex | 9.82 | -36 | 16 | 8 |
|  | R Inferior Frontal Gyrus, pars opercularis | 5.11 | 38 | 16 | 26 |
|  | L Subcallosal Cortex | 5.39 | -4 | 10 | -16 |
|  | R Temporal Pole | 9.17 | 52 | 10 | -4 |
|  | R Paracingulate Gyrus | 11.63 | 6 | 8 | 46 |
|  | R Insular Cortex | 10.17 | 38 | 8 | 4 |
|  | L Central Opercular Cortex | 8.74 | -46 | 6 | 2 |
|  | L Putamen | 7.03 | -26 | 6 | -8 |
|  | L Cingulate Gyrus, anterior | 11.73 | -8 | 6 | 40 |
|  | R Putamen | 7.26 | 24 | 6 | -8 |
|  | R Central Opercular Cortex | 8.95 | 46 | 6 | 0 |
|  | L Precentral Gyrus | 10.36 | -56 | 4 | 6 |
|  | L Superior Frontal Gyrus | 8.07 | -18 | 4 | 70 |
|  | R Cingulate Gyrus, anterior | 11.66 | 4 | 4 | 40 |
|  | R Bed Nucleus of the Stria Terminalis | 9.51 | 10 | 4 | 0 |
|  | L Juxtapositional Lobule Cortex (formerly Supplementary Motor Cortex) | 8.14 | -10 | 2 | 52 |
|  | L Bed Nucleus of the Stria Terminalis | 7.98 | -6 | 2 | -2 |
|  | R Juxtapositional Lobule Cortex | 10.86 | 8 | 2 | 50 |
|  | R Caudate | 8.33 | 10 | 2 | 8 |
|  | L Inferior Temporal Gyrus, anterior | 2.57 | -50 | 0 | -38 |
|  | L Temporal Fusiform Cortex, anterior | 2.98 | -32 | 0 | -42 |
|  | L Pallidum | 6.90 | -20 | 0 | 2 |
|  | R Superior Frontal Gyrus | 8.99 | 24 | 0 | 68 |
|  | R Middle Frontal/Precentral Gyri | 10.79 | 42 | 0 | 56 |
|  | R Inferior Temporal Gyrus, anterior | 2.86 | 46 | 0 | -46 |
|  | R Precentral Gyrus | 10.85 | 48 | 0 | 50 |
|  | L Middle Temporal Gyrus, anterior | 3.43 | -48 | -2 | -30 |
|  | L Middle Frontal Gyrus | 8.59 | -36 | -2 | 58 |
|  | R Pallidum | 8.97 | 12 | -2 | -6 |
|  | R Parahippocampal Gyrus, anterior | 3.83 | 24 | -2 | -34 |
|  | R Middle Temporal Gyrus, anterior | 3.77 | 50 | -2 | -28 |
|  | L Parahippocampal Gyrus, anterior | 3.41 | -20 | -4 | -36 |
|  | R Thalamus | 8.01 | 14 | -4 | 14 |
|  | L Caudate | 5.02 | -14 | -8 | 18 |
|  | R Planum Polare | 6.65 | 44 | -8 | -10 |
|  | R Heschl’s Gyrus | 6.37 | 46 | -8 | 0 |
|  | L Planum Polare | 6.35 | -44 | -16 | -2 |
|  | L Temporal Fusiform Cortex, posterior | 4.10 | -36 | -16 | -34 |
|  | L Thalamus | 5.39 | -6 | -16 | 16 |
|  | R Planum Temporale | 7.18 | 56 | -16 | 8 |
|  | R Postcentral Gyrus | 7.73 | 62 | -18 | 26 |
|  | L Heschl’s Gyrus | 6.51 | -50 | -20 | 6 |
|  | L Cingulate Gyrus, posterior | 8.30 | -2 | -20 | 28 |
|  | R Middle Temporal Gyrus, posterior | 6.78 | 62 | -20 | -4 |
|  | R Cingulate Gyrus, posterior | 9.00 | 6 | -22 | 44 |
|  | R Parietal Operculum Cortex | 10.41 | 52 | -22 | 16 |
|  | R Temporal Fusiform Cortex, posterior | 2.20 | 40 | -26 | -18 |
|  | L Postcentral Gyrus | 6.57 | -48 | -28 | 38 |
|  | L Middle Temporal Gyrus, posterior | 5.15 | -64 | -30 | 0 |
|  | R Brainstem | 9.64 | 4 | -30 | -4 |
|  | R Inferior Temporal Gyrus, posterior | 4.81 | 58 | -30 | -24 |
|  | R Supramarginal Gyrus, anterior | 9.93 | 58 | -30 | 36 |
|  | L Parietal Operculum Cortex | 8.55 | -62 | -32 | 20 |
|  | R Superior Temporal Gyrus, posterior | 8.19 | 66 | -32 | 12 |
|  | L Planum Temporale | 6.10 | -56 | -34 | 10 |
|  | R Hippocampus | 4.18 | 32 | -34 | -4 |
|  | L Supramarginal Gyrus, anterior | 7.96 | -60 | -36 | 28 |
|  | L Hippocampus | 4.68 | -20 | -36 | -4 |
|  | L Brainstem | 8.18 | -8 | -38 | -46 |
|  | R Supramarginal Gyrus, posterior | 9.86 | 62 | -40 | 20 |
|  | R Precuneus Cortex | 7.59 | 6 | -42 | 50 |
|  | L Supramarginal Gyrus, posterior | 9.57 | -52 | -44 | 40 |
|  | L Inferior Temporal Gyrus, posterior / Inferior Temporal Gyrus, temporooccipital | 4.18 | -50 | -44 | -24 |
|  | L Superior Parietal Lobule | 6.38 | -44 | -44 | 58 |
|  | R Angular Gyrus | 7.83 | 62 | -48 | 32 |
|  | L Inferior Temporal Gyrus, temporooccipital | 2.78 | -52 | -50 | -12 |
|  | L Precuneus Cortex | 6.91 | -10 | -50 | 54 |
|  | R Superior Parietal Lobule | 7.69 | 32 | -52 | 48 |
|  | L Middle Temporal Gyrus, temporooccipital /Angular Gyrus | 6.01 | -56 | -54 | 12 |
|  | L Angular Gyrus | 7.23 | -50 | -54 | 44 |
|  | R Inferior Temporal Gyrus, temporooccipital | 7.54 | 50 | -54 | -14 |
|  | L Temporal Occipital Fusiform Cortex | 10.51 | -30 | -56 | -18 |
|  | R Temporal Occipital Fusiform Cortex | 8.71 | 32 | -58 | -18 |
|  | R Middle Temporal Gyrus, temporooccipital /Lateral Occipital Cortex, inferior | 8.14 | 54 | -60 | 6 |
|  | L Lateral Occipital Cortex, superior | 5.79 | -12 | -62 | 54 |
|  | L Lateral Occipital Cortex, inferior | 6.61 | -44 | -64 | 8 |
|  | R Lateral Occipital Cortex, inferior | 8.16 | 38 | -78 | -10 |
|  | R Lateral Occipital Cortex, superior | 5.73 | 18 | -82 | 36 |
|  | R Occipital Fusiform Gyrus | 7.46 | 32 | -82 | -12 |
|  | L Occipital Fusiform Gyrus | 5.52 | -14 | -86 | -12 |
|  | L Occipital Pole | 6.03 | -36 | -94 | -6 |
|  | R Occipital Pole | 7.59 | 24 | -94 | 16 |
| 248 | L Temporal Pole | 3.00 | -26 | 8 | -46 |
| 216 | R Occipital Pole | 3.21 | 2 | -98 | 18 |
| 136 | R Subcallosal Cortex | 2.91 | 6 | 14 | -24 |
| 72 | L Frontal Pole | 3.06 | -40 | 62 | -2 |
| 48 | R Frontal Orbital Cortex | 2.02 | 16 | 20 | -24 |
| 48 | L Amygdala (Cortical Nucleus/Amygdalohippocampal Area)^a,b^ | 2.27 | -14 | -2 | -22 |
| 16 | L Frontal Pole | 1.97 | -36 | 44 | -16 |
| 16 | L Intracalcarine Cortex | 2.13 | -22 | -62 | 6 |
| 8 | R Temporal Pole | 2.03 | 22 | 16 | -42 |
| 8 | R Subcallosal/Frontal Orbital Cortices | 1.92 | 10 | 28 | -22 |
| 8 | L Frontal Pole | 1.96 | -10 | 64 | -22 |
| 8 | L Postcentral Gyrus | 2.94 | -4 | -46 | 72 |
| 8 | L Precentral Gyrus | 2.23 | -20 | -24 | 78 |

^a^Within the Harvard-Oxford amygdala (*p*>.25), a total of 173 2-mm^3^ voxels (1,384 mm^3^) exceeded threshold. ^b^ 38% probability of lying within the Harvard-Oxford amygdala.
