# Supplementary material for: Acute nicotine abstinence amplifies subjective withdrawal symptoms and threat-evoked fear and anxiety, but not extended amygdala reactivity
Source: PLoS One. 2023 Jul 20;18(7):e0288544. doi: 10.1371/journal.pone.0288544 (PMC10358993; doi:10.1371/journal.pone.0288544)
Supplement: S4 Table — (DOCX) [file pone.0288544.s005.docx]

**Acute nicotine abstinence amplifies subjective withdrawal symptoms and threat-evoked fear and anxiety, but not extended amygdala reactivity**

Hyung Cho Kim^1,2^

Claire M. Kaplan^4^

Samiha Islam^5^

Allegra S. Anderson^6^

Megan E. Piper^7^

Daniel E. Bradford^8^

John J. Curtin^9^

Kathryn A. DeYoung^1^

Jason F. Smith^1^

Andrew S. Fox^10,11^

Alexander J. Shackman^1,2,3^

^1^Department of Psychology, University of Maryland, College Park, Maryland, United States of America

^2^Neuroscience and Cognitive Science Program, University of Maryland, College Park, Maryland, United States of America

^3^Maryland Neuroimaging Center, University of Maryland, College Park, Maryland, United States of America

^4^Department of Psychiatry and Behavioral Sciences, School of Medicine, Johns Hopkins University, Baltimore, Maryland, United States of America

^5^Department of Psychology, University of Pennsylvania, Philadelphia, Pennsylvania, United States of America

^6^Department of Psychological Sciences, Vanderbilt University, Nashville, Tennessee, United States of America

^7^Center for Tobacco Research and Intervention and Department of Medicine, School of Medicine and Public Health, University of Wisconsin—Madison, Madison, Wisconsin, United States of America

^8^School of Psychological Sciences, Oregon State University, Corvallis, Oregon, United States of America

^9^Department of Psychology, University of Wisconsin—Madison, Madison, Wisconsin, United States of America

^10^Department of Psychology, University of California, Davis, California, United States of America

^11^California National Primate Research Center, University of California, Davis, California, United States of America

Corresponding author(s)

E-mail: [hkim1230@umd.edu](mailto:hkim1230@umd.edu) (HCK), E-mail: [shackman@umd.edu](mailto:shackman@umd.edu) (AJS)**Supplementary Table S4. Descriptive statistics for clusters and local extrema showing greater activity during the anticipation of Uncertain Safety compared to Uncertain Threat (FDR *q*<.05, whole-brain corrected).**

| **mm^3^** | **Label** | ***t*** | ***x*** | ***y*** | ***z*** |
| --- | --- | --- | --- | --- | --- |
| 20,384 | L Precuneus Cortex | 4.92 | -14 | -54 | 6 |
|  | L Lingual Gyrus | 5.40 | -6 | -62 | 6 |
|  | R Precuneus Cortex | 4.92 | 0 | -66 | 20 |
|  | R Lingual Gyrus/ Intracalcarine Cortex | 8.96 | 0 | -74 | 8 |
|  | L Intracalcarine Cortex | 9.88 | -12 | -78 | 8 |
|  | R Intracalcarine Cortex | 10.35 | 14 | -80 | 12 |
| 1,056 | R Frontal Pole | 4.67 | 2 | 58 | -12 |
|  | R Frontal Medial Cortex/Frontal Pole | 4.62 | 2 | 54 | -12 |
| 880 | R Precentral Gyrus | 5.10 | 4 | -28 | 64 |
| 416 | R Frontal Medial Cortex | 4.56 | 0 | 40 | -26 |
| 120 | R Lingual Gyrus | 3.89 | 30 | -52 | -4 |
| 88 | L Parahippocampal Gyrus, posterior | 4.10 | -30 | -38 | -12 |
| 64 | L Postcentral Gyrus | 3.83 | -54 | -20 | 58 |
| 56 | R Middle Temporal Gyrus, anterior | 3.85 | 64 | -2 | -22 |
| 40 | L Cingulate Gyrus, anterior | 3.89 | -4 | 34 | 4 |
| 24 | R Postcentral Gyrus | 3.70 | 56 | -18 | 56 |
| 16 | L Middle Temporal Gyrus, anterior | 3.46 | -62 | -4 | -18 |
| 8 | L Frontal Orbital Cortex | 3.42 | -34 | 34 | -10 |
| 8 | L Subcallosal Cortex | 3.49 | -4 | 28 | -6 |
