# Supplementary material for: Acute nicotine abstinence amplifies subjective withdrawal symptoms and threat-evoked fear and anxiety, but not extended amygdala reactivity
Source: PLoS One. 2023 Jul 20;18(7):e0288544. doi: 10.1371/journal.pone.0288544 (PMC10358993; doi:10.1371/journal.pone.0288544)
Supplement: S6 Table — (DOCX) [file pone.0288544.s007.docx]

**Acute nicotine abstinence amplifies subjective withdrawal symptoms and threat-evoked fear and anxiety, but not extended amygdala reactivity**

Hyung Cho Kim^1,2^

Claire M. Kaplan^4^

Samiha Islam^5^

Allegra S. Anderson^6^

Megan E. Piper^7^

Daniel E. Bradford^8^

John J. Curtin^9^

Kathryn A. DeYoung^1^

Jason F. Smith^1^

Andrew S. Fox^10,11^

Alexander J. Shackman^1,2,3^

^1^Department of Psychology, University of Maryland, College Park, Maryland, United States of America

^2^Neuroscience and Cognitive Science Program, University of Maryland, College Park, Maryland, United States of America

^3^Maryland Neuroimaging Center, University of Maryland, College Park, Maryland, United States of America

^4^Department of Psychiatry and Behavioral Sciences, School of Medicine, Johns Hopkins University, Baltimore, Maryland, United States of America

^5^Department of Psychology, University of Pennsylvania, Philadelphia, Pennsylvania, United States of America

^6^Department of Psychological Sciences, Vanderbilt University, Nashville, Tennessee, United States of America

^7^Center for Tobacco Research and Intervention and Department of Medicine, School of Medicine and Public Health, University of Wisconsin—Madison, Madison, Wisconsin, United States of America

^8^School of Psychological Sciences, Oregon State University, Corvallis, Oregon, United States of America

^9^Department of Psychology, University of Wisconsin—Madison, Madison, Wisconsin, United States of America

^10^Department of Psychology, University of California, Davis, California, United States of America

^11^California National Primate Research Center, University of California, Davis, California, United States of America

Corresponding author(s)

E-mail: [hkim1230@umd.edu](mailto:hkim1230@umd.edu) (HCK), E-mail: [shackman@umd.edu](mailto:shackman@umd.edu) (AJS)

**Supplementary Table S6. Descriptive statistics for clusters and local extrema showing greater activity during the anticipation of Certain Safety compared to Certain Threat (FDR *q*<.05, whole-brain corrected).**

| **mm^3^** | **Label** | ***t*** | ***x*** | ***y*** | ***z*** |
| --- | --- | --- | --- | --- | --- |
| 59,448 | R Parahippocampal Gyrus, posterior division | 5.08 | 26 | -30 | -20 |
|  | L Temporal Occipital Fusiform Cortex | 3.51 | -32 | -48 | -6 |
|  | R Cingulate Gyrus, posterior division | 4.10 | 4 | -48 | 34 |
|  | R Temporal Occipital Fusiform Cortex/Lingual Gyrus | 6.73 | 26 | -50 | -8 |
|  | R Precuneus Cortex | 4.88 | 4 | -54 | 16 |
|  | L Precuneus Cortex | 5.09 | -6 | -58 | 14 |
|  | R Lingual Gyrus | 7.05 | 26 | -58 | -8 |
|  | L Occipital Fusiform Gyrus | 5.73 | -26 | -68 | -8 |
|  | R Occipital Fusiform Gyrus | 6.22 | 28 | -68 | -8 |
|  | L Lingual Gyrus/ Intracalcarine Cortex | 9.39 | -2 | -78 | 2 |
|  | L Intracalcarine Cortex | 10.28 | -12 | -84 | 2 |
|  | R Intracalcarine Cortex | 12.82 | 10 | -86 | 4 |
|  | R Occipital Pole | 12.57 | 6 | -90 | 6 |
|  | L Occipital Pole | 10.14 | -4 | -94 | 4 |
| 2,736 | R Frontal Pole | 4.25 | 0 | 62 | -4 |
|  | L Frontal Medial Cortex | 3.92 | -6 | 54 | -12 |
|  | R Frontal Medial Cortex | 4.24 | 2 | 38 | -16 |
| 784 | R Cingulate Gyrus, anterior | 5.30 | 0 | 36 | -4 |
| 744 | R Superior Frontal Gyrus | 3.95 | 22 | 32 | 48 |
|  | R Middle Frontal Gyrus | 3.92 | 28 | 20 | 50 |
| 264 | L Frontal Pole | 4.20 | -10 | 68 | 2 |
| 232 | R Middle Temporal Gyrus, anterior | 3.51 | 62 | -4 | -18 |
|  | R Middle Temporal Gyrus, posterior | 3.50 | 54 | -10 | -16 |
| 96 | L Cingulate Gyrus, posterior | 4.15 | -10 | -44 | 6 |
| 80 | L Occipital Pole | 3.54 | -30 | -92 | 22 |
| 80 | R Lateral Occipital Cortex, superior | 3.43 | 46 | -68 | 32 |
| 80 | R Postcentral Gyrus | 3.25 | 2 | -36 | 60 |
| 56 | R Frontal Pole | 3.26 | 52 | 46 | -8 |
| 48 | L Parahippocampal Gyrus, posterior | 3.67 | -30 | -28 | -20 |
| 48 | R Occipital Pole | 3.54 | 8 | -90 | 36 |
| 32 | L Parahippocampal Gyrus, anterior | 3.38 | -18 | -22 | -24 |
| 24 | L Hippocampus | 3.31 | -34 | -32 | -8 |
| 24 | L Lateral Occipital Cortex, superior | 3.14 | -48 | -70 | 30 |
| 16 | L Temporal Pole | 3.14 | -36 | 12 | -28 |
| 16 | L Frontal Orbital Cortex/ Frontal Pole | 3.21 | -32 | 34 | -16 |
| 16 | L Paracingulate Gyrus | 3.22 | -8 | 38 | -10 |
| 16 | L Lateral Occipital Cortex, superior | 3.23 | -36 | -84 | 38 |
| 8 | R Temporal Pole | 3.10 | 38 | 20 | -40 |
| 8 | R Brainstem | 3.03 | 6 | -20 | -32 |
| 8 | L Parahippocampal Gyrus, posterior | 3.10 | -22 | -32 | -18 |
| 8 | L Parahippocampal Gyrus, posterior | 3.22 | -20 | -34 | -16 |
| 8 | L Occipital Pole | 3.02 | -2 | -92 | 32 |
| 8 | L Lateral Occipital Cortex, superior | 3.19 | -30 | -80 | 48 |
