# Supplementary material for: Acute nicotine abstinence amplifies subjective withdrawal symptoms and threat-evoked fear and anxiety, but not extended amygdala reactivity
Source: PLoS One. 2023 Jul 20;18(7):e0288544. doi: 10.1371/journal.pone.0288544 (PMC10358993; doi:10.1371/journal.pone.0288544)
Supplement: S7 Table — (DOCX) [file pone.0288544.s008.docx]

**Acute nicotine abstinence amplifies subjective withdrawal symptoms and threat-evoked fear and anxiety, but not extended amygdala reactivity**

Hyung Cho Kim^1,2^

Claire M. Kaplan^4^

Samiha Islam^5^

Allegra S. Anderson^6^

Megan E. Piper^7^

Daniel E. Bradford^8^

John J. Curtin^9^

Kathryn A. DeYoung^1^

Jason F. Smith^1^

Andrew S. Fox^10,11^

Alexander J. Shackman^1,2,3^

^1^Department of Psychology, University of Maryland, College Park, Maryland, United States of America

^2^Neuroscience and Cognitive Science Program, University of Maryland, College Park, Maryland, United States of America

^3^Maryland Neuroimaging Center, University of Maryland, College Park, Maryland, United States of America

^4^Department of Psychiatry and Behavioral Sciences, School of Medicine, Johns Hopkins University, Baltimore, Maryland, United States of America

^5^Department of Psychology, University of Pennsylvania, Philadelphia, Pennsylvania, United States of America

^6^Department of Psychological Sciences, Vanderbilt University, Nashville, Tennessee, United States of America

^7^Center for Tobacco Research and Intervention and Department of Medicine, School of Medicine and Public Health, University of Wisconsin—Madison, Madison, Wisconsin, United States of America

^8^School of Psychological Sciences, Oregon State University, Corvallis, Oregon, United States of America

^9^Department of Psychology, University of Wisconsin—Madison, Madison, Wisconsin, United States of America

^10^Department of Psychology, University of California, Davis, California, United States of America

^11^California National Primate Research Center, University of California, Davis, California, United States of America

Corresponding author(s)

E-mail: [hkim1230@umd.edu](mailto:hkim1230@umd.edu) (HCK), E-mail: [shackman@umd.edu](mailto:shackman@umd.edu) (AJS)

**Supplementary Table S7. Descriptive statistics for clusters and local extrema showing greater activity during the anticipation of Uncertain *and* Certain Threat (minimum conjunction of FDR-thresholded maps).**

| **mm^3^** | **Label** | ***t*** | ***x*** | ***y*** | ***z*** |
| --- | --- | --- | --- | --- | --- |
| 246,360 | L Frontal Orbital Cortex | 4.64 | -34 | 28 | -2 |
|  | R Inferior Frontal Gyrus, pars triangularis | 3.35 | 52 | 28 | 4 |
|  | R Frontal Orbital Cortex | 3.13 | 40 | 26 | -10 |
|  | L Frontal Operculum Cortex | 7.10 | -36 | 22 | 8 |
|  | L Insular/Frontal Orbital Cortices | 4.27 | -34 | 20 | -8 |
|  | R Frontal Operculum Cortex | 6.50 | 36 | 20 | 10 |
|  | L Cingulate Gyrus, anterior division | 6.04 | -4 | 18 | 34 |
|  | R Inferior Frontal Gyrus, pars opercularis | 4.39 | 48 | 16 | 24 |
|  | L Inferior Frontal Gyrus, pars opercularis | 3.26 | -56 | 12 | 16 |
|  | L Temporal Pole | 6.59 | -54 | 12 | -4 |
|  | R Temporal Pole | 4.64 | 56 | 12 | -8 |
|  | L Paracingulate Gyrus | 7.39 | -8 | 10 | 42 |
|  | R Paracingulate Gyrus | 6.74 | 4 | 10 | 54 |
|  | R Superior Frontal Gyrus | 5.52 | 12 | 10 | 70 |
|  | R Insular Cortex | 5.49 | 36 | 10 | 6 |
|  | R Cingulate Gyrus, anterior | 6.02 | 4 | 8 | 38 |
|  | R Putamen | 6.15 | 22 | 8 | -10 |
|  | R Central Opercular Cortex | 5.73 | 42 | 8 | 6 |
|  | L Accumbens | 3.42 | -8 | 6 | -8 |
|  | R Caudate | 4.65 | 16 | 6 | 16 |
|  | L Central Opercular Cortex | 6.11 | -46 | 4 | 2 |
|  | L Juxtapositional Lobule Cortex (formerly Supplementary Motor Cortex) | 6.19 | -10 | 4 | 50 |
|  | L Putamen | 5.47 | -28 | 2 | -6 |
|  | L Superior Frontal Gyrus | 5.70 | -16 | 2 | 68 |
|  | R Bed Nucleus of the Stria Terminalis | 5.99 | 10 | 2 | 2 |
|  | L Thalamus | 4.03 | -10 | -2 | 8 |
|  | R Juxtapositional Lobule Cortex (formerly Supplementary Motor Cortex) | 4.09 | 8 | -4 | 66 |
|  | R Precentral Gyrus | 6.16 | 44 | -4 | 48 |
|  | L Precentral Gyrus | 5.38 | -46 | -6 | 44 |
|  | R Thalamus | 5.21 | 12 | -8 | 0 |
|  | R Pallidum | 5.09 | 26 | -8 | -4 |
|  | L Pallidum | 3.15 | -18 | -10 | -4 |
|  | L Amygdala (Central/Medial Nuclei)^a,b^ | 3.36 | -26 | -12 | -12 |
|  | R Postcentral Gyrus | 3.59 | 44 | -20 | 40 |
|  | L Cingulate Gyrus, posterior | 4.40 | -4 | -24 | 26 |
|  | L Postcentral Gyrus | 3.16 | -44 | -26 | 40 |
|  | R Supramarginal Gyrus, anterior | 5.44 | 56 | -26 | 38 |
|  | L Brainstem | 4.31 | -6 | -28 | -8 |
|  | R Brainstem | 4.60 | 8 | -28 | -10 |
|  | R Cingulate Gyrus, posterior | 5.59 | 12 | -28 | 38 |
|  | R Supramarginal Gyrus, posterior | 4.28 | 54 | -38 | 28 |
|  | L Supramarginal Gyrus, anterior | 5.66 | -54 | -40 | 34 |
|  | L Supramarginal Gyrus, posterior | 4.77 | -56 | -44 | 20 |
|  | L Superior Parietal Lobule | 4.54 | -22 | -48 | 68 |
|  | L Precuneus Cortex | 4.69 | -12 | -50 | 56 |
|  | R Precuneus Cortex | 4.85 | 12 | -50 | 54 |
|  | R Superior Parietal Lobule | 4.87 | 22 | -50 | 58 |
|  | R Angular Gyrus | 2.85 | 52 | -50 | 36 |
|  | L Angular Gyrus | 3.11 | -52 | -54 | 46 |
|  | R Middle Temporal Gyrus, temporooccipital | 3.67 | 54 | -56 | 10 |
|  | L Lateral Occipital Cortex, superior | 4.22 | -16 | -84 | 36 |
|  | R Lateral Occipital Cortex, inferior | 3.74 | 44 | -88 | -4 |
|  | R Occipital Pole | 3.41 | 34 | -90 | 8 |
| 77,936 | L Brainstem | 3.12 | -6 | -32 | -44 |
|  | R Brainstem | 4.04 | 12 | -40 | -32 |
|  | L Lingual Gyrus | 5.29 | -4 | -74 | -12 |
| 12,008 | L Frontal Pole | 5.54 | -36 | 48 | 24 |
|  | L Middle Frontal Gyrus | 4.42 | -30 | 32 | 36 |
| 9,824 | R Frontal Pole | 6.60 | 34 | 50 | 34 |
| 3,064 | R Middle Temporal Gyrus, posterior | 4.14 | 50 | -36 | -4 |
|  | R Middle Temporal Gyrus, temporooccipital | 3.90 | 52 | -44 | 6 |
| 2,128 | R Precuneus Cortex | 3.29 | 18 | -70 | 34 |
|  | R Lateral Occipital Cortex, superior | 3.37 | 18 | -82 | 38 |
| 1,848 | R Heschl’s Gyrus | 3.26 | 46 | -20 | 8 |
|  | R Superior Temporal Gyrus, posterior | 2.48 | 66 | -30 | 10 |
| 1,488 | R Temporal Pole | 2.64 | 40 | 6 | -42 |
|  | R Middle Temporal Gyrus, anterior | 3.20 | 50 | 0 | -30 |
|  | R Parahippocampal Gyrus, anterior | 2.64 | 24 | -4 | -32 |
| 488 | L Middle Temporal Gyrus, temporooccipital | 2.99 | -50 | -54 | 4 |
| 312 | L Planum Temporale | 3.58 | -56 | -30 | 8 |
| 272 | L Brainstem | 3.10 | -6 | -44 | -54 |
| 240 | L Parahippocampal Gyrus, anterior | 3.01 | -20 | -2 | -38 |
| 176 | L Middle Temporal Gyrus, anterior | 2.70 | -48 | 0 | -30 |
| 160 | L Frontal Pole | 2.97 | -24 | 52 | -12 |
| 144 | L Middle Frontal Gyrus | 2.99 | -38 | 18 | 40 |
| 112 | R Planum Polare | 3.06 | 42 | -6 | -14 |
| 104 | L Temporal Pole | 2.79 | -38 | 2 | -40 |
| 88 | L Paracingulate Gyrus / Cingulate Gyrus, anterior | 2.99 | -6 | 40 | 20 |
| 80 | L Planum Polare | 2.90 | -42 | -2 | -18 |
| 80 | L Temporal Pole | 3.03 | -48 | 20 | -18 |
| 80 | L Precentral Gyrus | 3.11 | -38 | -2 | 30 |
| 72 | L Temporal Fusiform Cortex, anterior | 2.61 | -30 | -8 | -38 |
| 72 | R Thalamus | 2.81 | 6 | -18 | 14 |
| 64 | R Paracingulate Gyrus | 2.86 | 14 | 50 | 4 |
| 56 | L Inferior Temporal Gyrus, posterior | 2.77 | -56 | -28 | -30 |
| 48 | R Inferior Temporal Gyrus, posterior / Temporal Fusiform Cortex, posterior | 2.70 | 44 | -32 | -24 |
| 48 | R Postcentral Gyrus | 2.50 | 32 | -28 | 62 |
| 32 | R Frontal Pole | 2.43 | 20 | 70 | -8 |
| 32 | R Lateral Occipital Cortex, inferior | 2.46 | 40 | -70 | -6 |
| 32 | L Middle Temporal Gyrus, temporooccipital | 2.67 | -66 | -54 | -2 |
| 24 | L Temporal Fusiform Cortex, anterior | 2.39 | -26 | -6 | -42 |
| 24 | L Frontal Orbital Cortex | 2.63 | -20 | 22 | -20 |
| 24 | L Lateral Occipital Cortex, inferior | 2.55 | -56 | -74 | -2 |
| 16 | L Temporal Pole | 2.64 | -28 | 6 | -48 |
| 16 | L Temporal Pole | 1.98 | -32 | 18 | -36 |
| 16 | L Superior Parietal Lobule | 2.61 | -26 | -56 | 44 |
| 8 | R Parahippocampal Gyrus, anterior | 1.96 | 24 | 0 | -30 |
| 8 | L Inferior Temporal Gyrus, posterior | 2.11 | -56 | -34 | -22 |
| 8 | L Frontal Pole | 2.01 | -20 | 64 | -16 |

^a^Within the Harvard-Oxford amygdala (*p*>.25), a total of 30 2-mm^3^ voxels (240 mm^3^) exceeded threshold. ^b^57% probability of lying within the Harvard-Oxford amygdala.
