# Supplementary material for: Acute nicotine abstinence amplifies subjective withdrawal symptoms and threat-evoked fear and anxiety, but not extended amygdala reactivity
Source: PLoS One. 2023 Jul 20;18(7):e0288544. doi: 10.1371/journal.pone.0288544 (PMC10358993; doi:10.1371/journal.pone.0288544)
Supplement: S9 Table — (DOCX) [file pone.0288544.s010.docx]

**Acute nicotine abstinence amplifies subjective withdrawal symptoms and threat-evoked fear and anxiety, but not extended amygdala reactivity**

Hyung Cho Kim^1,2^

Claire M. Kaplan^4^

Samiha Islam^5^

Allegra S. Anderson^6^

Megan E. Piper^7^

Daniel E. Bradford^8^

John J. Curtin^9^

Kathryn A. DeYoung^1^

Jason F. Smith^1^

Andrew S. Fox^10,11^

Alexander J. Shackman^1,2,3^

^1^Department of Psychology, University of Maryland, College Park, Maryland, United States of America

^2^Neuroscience and Cognitive Science Program, University of Maryland, College Park, Maryland, United States of America

^3^Maryland Neuroimaging Center, University of Maryland, College Park, Maryland, United States of America

^4^Department of Psychiatry and Behavioral Sciences, School of Medicine, Johns Hopkins University, Baltimore, Maryland, United States of America

^5^Department of Psychology, University of Pennsylvania, Philadelphia, Pennsylvania, United States of America

^6^Department of Psychological Sciences, Vanderbilt University, Nashville, Tennessee, United States of America

^7^Center for Tobacco Research and Intervention and Department of Medicine, School of Medicine and Public Health, University of Wisconsin—Madison, Madison, Wisconsin, United States of America

^8^School of Psychological Sciences, Oregon State University, Corvallis, Oregon, United States of America

^9^Department of Psychology, University of Wisconsin—Madison, Madison, Wisconsin, United States of America

^10^Department of Psychology, University of California, Davis, California, United States of America

^11^California National Primate Research Center, University of California, Davis, California, United States of America

Corresponding author(s)

E-mail: [hkim1230@umd.edu](mailto:hkim1230@umd.edu) (HCK), E-mail: [shackman@umd.edu](mailto:shackman@umd.edu) (AJS)

**Supplementary Table S9. Spearman correlations among key outcome measures, all participants.**

| **All Participants** | **Smoking Urges/Withdrawal** | | | **Fear/Anxiety Ratings** | | | **Skin Conductance Level** | | | **fMRI: BST** | | | **fMRI: Ce** | | |
| --- | --- | --- | --- | --- | --- | --- | --- | --- | --- | --- | --- | --- | --- | --- | --- |
| **Spearman's Rho** | **BQSU** | **WSWS** | **WSWS_ANX** | **TmS** | **UTmUS** | **CTmCS** | **TmS** | **UTmUS** | **PTmPS** | **TmS** | **CTmCS** | **UTmUS** | **TmS** | **CTmCS** | **UTmUS** |
| **BQSU** | . |  |  |  |  |  |  |  |  |  |  |  |  |  |  |
| **WSWS** | **0.62** | . |  |  |  |  |  |  |  |  |  |  |  |  |  |
| **WSWS_ANX** | **0.51** | **0.87** | . |  |  |  |  |  |  |  |  |  |  |  |  |
| **RATING_TmS** | **0.40** | **0.36** | **0.36** | . |  |  |  |  |  |  |  |  |  |  |  |
| **RATING_UTmUS** | **0.42** | **0.36** | **0.34** | **0.91** | . |  |  |  |  |  |  |  |  |  |  |
| **RATING_CTmCS** | **0.29** | **0.28** | **0.30** | **0.89** | **0.61** | . |  |  |  |  |  |  |  |  |  |
| **SCR_TmS** | 0.09 | 0.05 | 0.02 | 0.18 | 0.13 | 0.19 | . |  |  |  |  |  |  |  |  |
| **SCR_UTmUS** | 0.14 | 0.05 | 0.06 | **0.34** | **0.28** | **0.33** | **0.84** | . |  |  |  |  |  |  |  |
| **SCR_CTmCS** | 0.08 | -0.18 | -0.21 | 0.01 | -0.07 | 0.10 | **0.62** | **0.35** | . |  |  |  |  |  |  |
| **BST_TmS** | 0.06 | 0.05 | -0.03 | 0.00 | -0.01 | 0.02 | **0.32** | 0.15 | 0.10 | . |  |  |  |  |  |
| **BST_CTmCS** | 0.11 | 0.04 | 0.02 | -0.09 | -0.06 | -0.10 | **0.28** | 0.04 | 0.18 | **0.71** | . |  |  |  |  |
| **BST_UTmUS** | -0.01 | 0.04 | -0.06 | 0.08 | 0.03 | 0.11 | 0.19 | 0.17 | -0.02 | **0.79** | 0.13 | . |  |  |  |
| **Ce_TmS** | 0.01 | -0.01 | -0.05 | 0.03 | 0.04 | 0.02 | 0.13 | 0.08 | 0.07 | 0.13 | 0.05 | 0.14 | . |  |  |
| **Ce_CTmCS** | -0.03 | 0.00 | 0.08 | -0.06 | -0.05 | -0.06 | 0.08 | 0.15 | -0.02 | -0.01 | 0.02 | -0.04 | **0.71** | . |  |
| **Ce_UTmUS** | 0.05 | 0.00 | -0.16 | 0.11 | 0.11 | 0.08 | 0.09 | -0.04 | 0.11 | 0.20 | 0.05 | 0.24 | **0.67** | -0.05 | . |
|  |  |  |  |  |  |  |  |  |  |  |  |  |  |  |  |
| **Nominal *p*** | **BQSU** | **WSWS** | **WSWS_ANX** | **TmS** | **UTmUS** | **CTmCS** | **TmS** | **UTmUS** | **PTmPS** | **TmS** | **CTmCS** | **UTmUS** | **TmS** | **CTmCS** | **UTmUS** |
| **BQSU** | . |  |  |  |  |  |  |  |  |  |  |  |  |  |  |
| **WSWS** | 0.00 | . |  |  |  |  |  |  |  |  |  |  |  |  |  |
| **WSWS_ANX** | 0.00 | 0.00 | . |  |  |  |  |  |  |  |  |  |  |  |  |
| **RATING_TmS** | 0.00 | 0.00 | 0.00 | . |  |  |  |  |  |  |  |  |  |  |  |
| **RATING_UTmUS** | 0.00 | 0.00 | 0.00 | 0.00 | . |  |  |  |  |  |  |  |  |  |  |
| **RATING_CTmCS** | 0.01 | 0.01 | 0.01 | 0.00 | 0.00 | . |  |  |  |  |  |  |  |  |  |
| **SCR_TmS** | 0.45 | 0.71 | 0.84 | 0.15 | 0.28 | 0.12 | . |  |  |  |  |  |  |  |  |
| **SCR_UTmUS** | 0.24 | 0.65 | 0.58 | 0.00 | 0.01 | 0.00 | 0.00 | . |  |  |  |  |  |  |  |
| **SCR_CTmCS** | 0.48 | 0.13 | 0.07 | 0.91 | 0.55 | 0.38 | 0.00 | 0.00 | . |  |  |  |  |  |  |
| **BST_TmS** | 0.60 | 0.66 | 0.78 | 0.99 | 0.92 | 0.88 | 0.01 | 0.21 | 0.41 | . |  |  |  |  |  |
| **BST_CTmCS** | 0.35 | 0.76 | 0.86 | 0.46 | 0.62 | 0.39 | 0.02 | 0.72 | 0.12 | 0.00 | . |  |  |  |  |
| **BST_UTmUS** | 0.94 | 0.73 | 0.58 | 0.50 | 0.77 | 0.34 | 0.12 | 0.14 | 0.86 | 0.00 | 0.28 | . |  |  |  |
| **Ce_TmS** | 0.94 | 0.96 | 0.66 | 0.79 | 0.74 | 0.89 | 0.29 | 0.48 | 0.57 | 0.25 | 0.65 | 0.22 | . |  |  |
| **Ce_CTmCS** | 0.77 | 0.98 | 0.50 | 0.60 | 0.64 | 0.63 | 0.50 | 0.20 | 0.87 | 0.92 | 0.85 | 0.76 | 0.00 | . |  |
| **Ce_UTmUS** | 0.67 | 0.97 | 0.18 | 0.35 | 0.34 | 0.48 | 0.45 | 0.73 | 0.33 | 0.08 | 0.66 | 0.04 | 0.00 | 0.65 | . |

Abbreviations—BST, bed nucleus of the stria terminalis; BQSU, Brief Questionnaire of Smoking Urges; Ce, central nucleus of the amygdala; CTmCS, Certain Threat minus Certain Safety anticipation; TmS, Threat minus Safety anticipation; UTmUS, Uncertain Threat minus Uncertain Safety anticipation; WSWS, Wisconsin Smoking Withdrawal Scale; WSWS_ANX, Wisconsin Smoking Withdrawal Scale, Anxiety Scale.
