# Supplementary material for: Acute nicotine abstinence amplifies subjective withdrawal symptoms and threat-evoked fear and anxiety, but not extended amygdala reactivity
Source: PLoS One. 2023 Jul 20;18(7):e0288544. doi: 10.1371/journal.pone.0288544 (PMC10358993; doi:10.1371/journal.pone.0288544)
Supplement: S10 Table — (DOCX) [file pone.0288544.s011.docx]

**Acute nicotine abstinence amplifies subjective withdrawal symptoms and threat-evoked fear and anxiety, but not extended amygdala reactivity**

Hyung Cho Kim^1,2^

Claire M. Kaplan^4^

Samiha Islam^5^

Allegra S. Anderson^6^

Megan E. Piper^7^

Daniel E. Bradford^8^

John J. Curtin^9^

Kathryn A. DeYoung^1^

Jason F. Smith^1^

Andrew S. Fox^10,11^

Alexander J. Shackman^1,2,3^

^1^Department of Psychology, University of Maryland, College Park, Maryland, United States of America

^2^Neuroscience and Cognitive Science Program, University of Maryland, College Park, Maryland, United States of America

^3^Maryland Neuroimaging Center, University of Maryland, College Park, Maryland, United States of America

^4^Department of Psychiatry and Behavioral Sciences, School of Medicine, Johns Hopkins University, Baltimore, Maryland, United States of America

^5^Department of Psychology, University of Pennsylvania, Philadelphia, Pennsylvania, United States of America

^6^Department of Psychological Sciences, Vanderbilt University, Nashville, Tennessee, United States of America

^7^Center for Tobacco Research and Intervention and Department of Medicine, School of Medicine and Public Health, University of Wisconsin—Madison, Madison, Wisconsin, United States of America

^8^School of Psychological Sciences, Oregon State University, Corvallis, Oregon, United States of America

^9^Department of Psychology, University of Wisconsin—Madison, Madison, Wisconsin, United States of America

^10^Department of Psychology, University of California, Davis, California, United States of America

^11^California National Primate Research Center, University of California, Davis, California, United States of America

Corresponding author(s)

E-mail: [hkim1230@umd.edu](mailto:hkim1230@umd.edu) (HCK), E-mail: [shackman@umd.edu](mailto:shackman@umd.edu) (AJS)

**Supplementary Table S10. Spearman correlations among key outcome measures, nicotine-abstinent participants.**

| **24-Hour Abstinence** | **Smoking Urges/Withdrawal** | | | **Fear/Anxiety Ratings** | | | **Skin Conductance Level** | | | **fMRI: BST** | | | **fMRI: Ce** | | |
| --- | --- | --- | --- | --- | --- | --- | --- | --- | --- | --- | --- | --- | --- | --- | --- |
| **Spearman's Rho** | **BQSU** | **WSWS** | **WSWS_ANX** | **TmS** | **UTmUS** | **CTmCS** | **TmS** | **UTmUS** | **PTmPS** | **TmS** | **CTmCS** | **UTmUS** | **TmS** | **CTmCS** | **UTmUS** |
| **BQSU** | . |  |  |  |  |  |  |  |  |  |  |  |  |  |  |
| **WSWS** | **0.44** | . |  |  |  |  |  |  |  |  |  |  |  |  |  |
| **WSWS_ANX** | **0.42** | **0.90** | . |  |  |  |  |  |  |  |  |  |  |  |  |
| **RATING_TmS** | 0.05 | 0.16 | 0.13 | . |  |  |  |  |  |  |  |  |  |  |  |
| **RATING_UTmUS** | -0.03 | 0.08 | 0.10 | **0.89** | . |  |  |  |  |  |  |  |  |  |  |
| **RATING_CTmCS** | 0.11 | 0.21 | 0.13 | **0.92** | **0.64** | . |  |  |  |  |  |  |  |  |  |
| **SCR_TmS** | -0.02 | -0.07 | -0.11 | 0.13 | 0.06 | 0.18 | . |  |  |  |  |  |  |  |  |
| **SCR_UTmUS** | -0.08 | 0.07 | 0.06 | 0.38 | **0.35** | **0.35** | **0.80** | . |  |  |  |  |  |  |  |
| **SCR_CTmCS** | 0.10 | -0.25 | -0.30 | 0.03 | -0.07 | 0.11 | **0.65** | **0.32** | . |  |  |  |  |  |  |
| **BST_TmS** | 0.02 | -0.02 | 0.04 | 0.13 | 0.06 | 0.16 | **0.45** | 0.26 | 0.15 | . |  |  |  |  |  |
| **BST_CTmCS** | 0.03 | -0.15 | -0.02 | -0.11 | -0.14 | -0.07 | 0.27 | 0.12 | 0.26 | **0.69** | . |  |  |  |  |
| **BST_UTmUS** | 0.00 | 0.10 | 0.07 | 0.26 | 0.19 | 0.28 | **0.40** | 0.26 | 0.00 | **0.81** | 0.14 | . |  |  |  |
| **Ce_TmS** | **-0.33** | -0.02 | -0.03 | -0.10 | -0.08 | -0.09 | 0.20 | 0.03 | -0.17 | 0.11 | 0.18 | 0.01 | . |  |  |
| **Ce_CTmCS** | **-0.32** | 0.00 | 0.12 | -0.22 | -0.20 | -0.21 | -0.07 | -0.07 | -0.22 | -0.15 | 0.12 | -0.31 | **0.69** | . |  |
| **Ce_UTmUS** | -0.10 | -0.02 | -0.18 | 0.12 | 0.11 | 0.10 | **0.36** | 0.12 | 0.02 | **0.32** | 0.11 | **0.35** | **0.61** | -0.15 | . |
|  |  |  |  |  |  |  |  |  |  |  |  |  |  |  |  |
| **Nominal *p*** | **BQSU** | **WSWS** | **WSWS_ANX** | **TmS** | **UTmUS** | **CTmCS** | **TmS** | **UTmUS** | **PTmPS** | **TmS** | **CTmCS** | **UTmUS** | **TmS** | **CTmCS** | **UTmUS** |
| **BQSU** | . |  |  |  |  |  |  |  |  |  |  |  |  |  |  |
| **WSWS** | 0.01 | . |  |  |  |  |  |  |  |  |  |  |  |  |  |
| **WSWS_ANX** | 0.01 | 0.00 | . |  |  |  |  |  |  |  |  |  |  |  |  |
| **RATING_TmS** | 0.77 | 0.32 | 0.44 | . |  |  |  |  |  |  |  |  |  |  |  |
| **RATING_UTmUS** | 0.84 | 0.62 | 0.53 | 0.00 | . |  |  |  |  |  |  |  |  |  |  |
| **RATING_CTmCS** | 0.50 | 0.21 | 0.44 | 0.00 | 0.00 | . |  |  |  |  |  |  |  |  |  |
| **SCR_TmS** | 0.92 | 0.68 | 0.52 | 0.44 | 0.75 | 0.30 | . |  |  |  |  |  |  |  |  |
| **SCR_UTmUS** | 0.62 | 0.70 | 0.73 | 0.02 | 0.03 | 0.03 | 0.00 | . |  |  |  |  |  |  |  |
| **SCR_CTmCS** | 0.54 | 0.13 | 0.07 | 0.88 | 0.66 | 0.51 | 0.00 | 0.05 | . |  |  |  |  |  |  |
| **BST_TmS** | 0.91 | 0.92 | 0.81 | 0.45 | 0.72 | 0.33 | 0.01 | 0.12 | 0.36 | . |  |  |  |  |  |
| **BST_CTmCS** | 0.85 | 0.38 | 0.93 | 0.50 | 0.41 | 0.67 | 0.11 | 0.49 | 0.12 | 0.00 | . |  |  |  |  |
| **BST_UTmUS** | 1.00 | 0.57 | 0.69 | 0.11 | 0.24 | 0.09 | 0.02 | 0.11 | 0.99 | 0.00 | 0.39 | . |  |  |  |
| **Ce_TmS** | 0.04 | 0.93 | 0.84 | 0.57 | 0.65 | 0.57 | 0.25 | 0.86 | 0.32 | 0.50 | 0.28 | 0.96 | . |  |  |
| **Ce_CTmCS** | 0.05 | 1.00 | 0.48 | 0.18 | 0.24 | 0.21 | 0.67 | 0.66 | 0.18 | 0.36 | 0.46 | 0.06 | 0.00 | . |  |
| **Ce_UTmUS** | 0.56 | 0.90 | 0.29 | 0.49 | 0.50 | 0.56 | 0.03 | 0.47 | 0.91 | 0.05 | 0.50 | 0.03 | 0.00 | 0.36 | . |

Abbreviations—BST, bed nucleus of the stria terminalis; BQSU, Brief Questionnaire of Smoking Urges; Ce, central nucleus of the amygdala; CTmCS, Certain Threat minus Certain Safety anticipation; TmS, Threat minus Safety anticipation; UTmUS, Uncertain Threat minus Uncertain Safety anticipation; WSWS, Wisconsin Smoking Withdrawal Scale; WSWS_ANX, Wisconsin Smoking Withdrawal Scale, Anxiety Scale.
